# Psychological Impact of COVID-19 in the Setting of Dentistry: A Review Article

**DOI:** 10.3390/ijerph192316216

**Published:** 2022-12-04

**Authors:** Juan Carlos De Haro, Eva María Rosel, Inmaculada Salcedo-Bellido, Ester Leno-Durán, Pilar Requena, Rocío Barrios-Rodríguez

**Affiliations:** 1 Faculty of Dentistry, University of Granada, 18011 Granada, Spain; 2 Departamento de Estomatología, Facultad de Odontología, Universidad de Granada, 18011 Granada, Spain; 3 Departamento de Medicina Preventiva y Salud Pública, Universidad de Granada, 18011 Granada, Spain; 4 Consortium for Biomedical Research in Epidemiology and Public Health (CIBERESP), 28029 Madrid, Spain; 5 Instituto de Investigación Biosanitaria (ibs. GRANADA), 18014 Granada, Spain; 6 Departamento de Obstetricia y Ginecología, Universidad de Granada, 18011 Granada, Spain

**Keywords:** COVID-19, dentistry, patients, psychological factors

## Abstract

The worldwide pandemic has exposed healthcare professionals to a high risk of infection, exacerbating the situation of uncertainty caused by COVID-19. The objective of this review was to evaluate the psychological impact of the COVID-19 pandemic on dental professionals and their patients. A literature review was conducted using Medline-Pubmed, Web of Science, and Scopus databases, excluding systematic reviews, narratives, meta-analyses, case reports, book chapters, short communications, and congress papers. A modified version of the Newcastle-Ottawa Scale (NOS) was used to evaluate the quality of the selected studies. The search retrieved 3879 articles, and 123 of these were selected for the review (7 longitudinal and 116 cross-sectional studies). Elevated anxiety levels were observed in dental professionals, especially in younger and female professionals. Except for orthodontic treatments, patients reported a high level of fear that reduced their demand for dentist treatment to emergency cases alone. The results suggest that the COVID-19 pandemic has had psychological and emotional consequences for dental professionals and their patients. Further research is necessary to evaluate the persistence of this problem over time.

## 1. Introduction

COVID-19 produced a state of generalized fear that has been studied in various social settings reviewed in [1]. The risk of infection affects the whole population but is greater among healthcare workers due to their frequent close contact with infected symptomatic or asymptomatic individuals [2], making them especially vulnerable to the impact of the pandemic [3].

Among healthcare professionals, dentists in particular have had to introduce numerous modifications in their daily clinical practice [4]. COVID-19 is known to be transmitted via aerosols and droplets [5], to which dentists are exposed in oral interventions, when they are in close proximity to the oropharynx of the patient [6]. These circumstances have increased the work stress levels of dentists and their fear of infecting family members. Some countries almost completely halted activity in dental clinics during certain phases of the pandemic [7]. These restrictions and changes have exacerbated feelings of so-called “dental phobia” that can often be responsible for delaying or avoiding non-emergency treatments [8]. Such delays can lead to dental emergencies that require procedures carrying a greater risk of infection by SARS-CoV-2 [9].

Since the beginning of 2020, numerous studies have investigated the psychological disorders produced by this situation in dental professionals and their patients. Two systematic reviews have addressed this issue [10,11], but they were limited to the first few months of the pandemic and only included articles studying the psychological impact on dental professionals. An updated review is warranted to include the numerous studies published since their publication and which also address the effect on the patients.

With this background, the objective of this study was to review studies on the psychological consequences of the COVID-19 pandemic for dental professionals and patients as well as the factors associated with the psychological impact.

## 2. Materials and Methods

### 2.1. Search Strategy and Selection Criteria

The search of this review was conducted in Medline-PubMed, Web of Science, and Scopus databases. The objective of this review was to address the question: “What emotional consequences has the COVID-19 pandemic had for dental professionals and their patients?”. The search strategy was: (COVID-19 OR COVID19 OR SARS-CoV-2 OR SARS OR Coronavirus OR Corona OR 2019-nCoV OR 2019-new coronavirus OR 2019-novel coronavirus OR Pandemic) AND (Dentistry OR Dentists OR Dental OR Dental Care OR Dental Patients) AND (Psychology OR Mental Health OR Fear OR Anxiety OR Distress OR Burnout OR Stress OR Depression OR Insomnia OR Psychiatry).

The following review inclusion criteria were established: (1) studies analyzing anxiety, fear, or stress caused by the COVID-19 pandemic in dentists and/or their patients; (2) studies written in English, Italian, or Spanish; and (3) studies published between 1 December 2019 and 1 January 2022. Exclusion criteria were: (1) systematic reviews, narratives, or meta-analyses; and (2) case reports, book chapters, short communications, and congress communications.

After eliminating duplicates, the titles and abstracts of retrieved articles were screened to exclude non-eligible items. The whole text of the remaining articles was then reviewed to establish their eligibility for the review. A reverse search of reference lists from all the relevant original articles and previous systematic reviews and meta-analyses was also done.

### 2.2. Data Extraction and Quality Evaluation

The following data were gathered from each article: (1) first author and year of publication; (2) country of study population; (3) sample size; (4) professional category/treatment undergone by patients; (5) demographic characteristics of the sample; (6) variables of interest analyzed and sources of information; and (7) main results.

The Newcastle-Ottawa Scale (NOS) was used to evaluate the quality of the reviewed studies [12], assigning a score (stars) based on three quality parameters: sample selection, comparability, and results evaluation. Studies are classified as: very good quality (9–10 stars), good (7–8 stars), satisfactory (5–6 stars), or unsatisfactory (<5 stars). This scale was originally designed for longitudinal studies but it has been modified to evaluate cross-sectional studies in previous studies [13,14]. We have done an additional adaptation for this study: the point 4 of ‘Selection’ section is referred to the validity of measurement tool. The studies selected in this review used the tools to evaluate the psychological impact as a result (consequence of the pandemic) and not as an exposure. So, this point was moved to ‘Outcome’ section (“Assessment of the outcome II”). Nevertheless, the final score remained unchanged (Supplementary Materials S1).

## 3. Results

**Figure 1:**
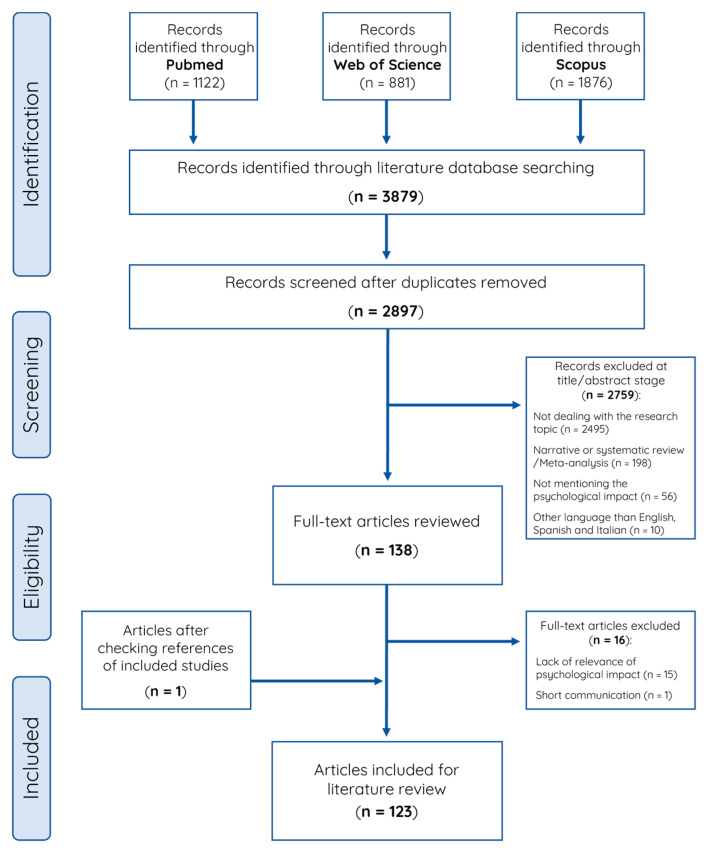
Literature review flow chart.

The search strategy retrieved 3879 articles, of which 982 were excluded as duplicates. Titles and abstracts of the remaining 2897 articles were then reviewed, and 2759 were excluded. After reading the whole text of the remaining 138 articles, 16 were excluded and a further article was added by reverse search (Figure 1). Finally, 123 studies were included in the review, 80 focusing on dental professionals [15,16,17,18,19,20,21,22,23,24,25,26,27,28,29,30,31,32,33,34,35,36,37,38,39,40,41,42,43,44,45,46,47,48,49,50,51,52,53,54,55,56,57,58,59,60,61,62,63,64,65,66,67,68,69,70,71,72,73,74,75,76,77,78,79,80,81,82,83,84,85,86,87,88,89,90,91,92,93,94] and 43 on patients [95,96,97,98,99,100,101,102,103,104,105,106,107,108,109,110,111,112,113,114,115,116,117,118,119,120,121,122,123,124,125,126,127,128,129,130,131,132,133,134,135,136,137].

### 3.1. Characteristics of Studies

The main characteristics and results of the studies are summarized in Table 1, Table 2, Table 3 and Table 4, which have been divided according to the use of validated questionnaires (Table 1 in professionals and Table 3 in patients) and non-validated ones (Table 2 in professionals and Table 4 in patients). The percentage of studies using validated questionnaires was 52.5% in professionals and 55.8% in patients.

All except for seven longitudinal studies [30,48,55,61,110,121,127] had a cross-sectional design. Online questionnaires served as the source of information in 107 of the 116 cross-sectional studies. The year of publication was 2020 in 33 studies (26.8%). The research was most frequently conducted in India (n = 20), followed by Turkey (n = 13), Saudi Arabia (n = 12), Italy (n = 11), and Brazil (n = 10) [Table S1 for further information]. The psychological aspects most frequently evaluated were anxiety (n = 58), fear (n = 38), stress (n = 30), and depression (n = 22). The sample size ranged between 15 and 5170 individuals. Patients were most frequently in the 30–50-year age group; there was a slight predominance of female sex in 86 studies, and the majority of dental professionals worked in a private practice.

Comparing the results of the studies using validated and non-validated questionnaires, there were differences in the percentage of dentists suffering from anxiety, with higher frequencies in those studies carried out with non-validated questionnaires (ranging from 7.1 to 71% with validated questionnaires [71,83] vs. 25.6 to 89% with non-validated questionnaires [64,81]). This was also observed in the studies that analysed adult patients under general treatment (ranging from 4.5 to 5.1% with validated questionnaires [100,108] vs. 9.5 to 62.4% with non-validated questionnaires [76,133]). Similarly, studies analyzing the percentage of patients presenting fear reported higher values with validated questionnaires (from 45 to 45.7% [100,120]) than with the non-validated ones (62.4 to 63.6% [115,133]).

The data collection period of the studies covered from November 2019 to July 2021, and the pandemic moment in each one of the countries [138] is summarized in Supplementary Tables S2 and S3. The vaccination process was not initiated in most part of the studies by the time of data collection. The pandemic situation was instead very different within the studies, with some recruiting participants during the first and second waves, and therefore at times of high rate of cases and deaths, and some of them recruiting at timings of low incidence of COVID-19 [138].

### 3.2. Quality Evaluation

Supplementary Materials, Tables S4 and S5 exhibit the NOS scores assigned to the studies: 10 studies (8.1%) had very good quality (9 stars), 52 (42.3%) good (7–8 stars), 56 (45.5%) satisfactory (5–6 stars), and only 5 (4.1%) unsatisfactory (4 stars). The main study limitations were the failure to calculate the required sample size and the lack of control for potential confounders in the data gathering or results analysis.

### 3.3. Impact of COVID-19 on Professionals

The professionals under study were: general dentists in 70 studies [15,16,17,18,20,21,22,23,24,25,26,27,28,29,30,31,32,33,34,37,39,40,41,42,43,44,45,46,47,48,49,50,53,54,55,56,57,58,62,63,64,65,66,67,68,69,70,71,72,73,74,75,76,77,78,79,80,81,82,83,84,85,86,87,88,89,90,92,93,94], some of which also included dental assistants or hygienists; orthodontists in 3 studies [51,60,91]; endodontists in 2 [59,61]; pediatric dentists in 1 [38]; and dental assistants/hygienists alone in 4 [19,35,36,52].

The main study variable in investigations on general dentists was anxiety (n = 35). Elevated anxiety levels about the possible contraction of COVID-19 from patients was observed in up to 89% of professionals [81], ranging from 1.7 to 23% those reporting severe anxiety [29,42]. Distrust about the effectiveness of protective measures and equipment during the first phase of the pandemic was expressed by 83.1% of professionals [84], and clinical activity was suspended at some point by 71.2% [86]. The subsequent resumption of activity was associated with increased anxiety levels, especially in professionals performing procedures with high aerosol generation [48]. Various studies found that a higher anxiety level was significantly associated with younger age and female sex of the dentist [28,36,43,48,50,52].

Other psychological factors studied included depression (n = 15), stress (n = 22), distress (n = 6), and burnout (n = 4). Depression-related symptoms were found in up to 60% of general dentists [71], having severe depression up to 22% [27]. Fear of infection and perceived work insecurity were positively associated with more depressive symptoms [39,83] and with the presence of some type of underlying disease [29,53,83]. Stress had a prevalence of up to 92% in professionals [71] and the severe stress ranged from 0.7 to 45% [29,71]. It was observed that stress was significantly increased among dentists during the first few weeks of the pandemic (from 18.61 ± 6.87 to 20.72 ± 1.95 on a 40-point scale; *p* < 0.0001) [55]. Clinical symptoms of post-traumatic stress disorder were recorded in 1.1 up to 32.3% of professionals [29,40]. Mild-severe distress was observed in 11.5–57.8% of general dentists [31,79], with a higher prevalence among females and under 40-year-olds [58]. Other distress-related factors were the presence of underlying disease, fear of infection by patients, and work overload [79], with 55.6% of professionals describing states of emotional exhaustion [40].

Studies of orthodontists mainly evaluated anxiety (n = 2), which was almost five-fold more frequent among those working in public versus private settings (60% vs. 12.6%, respectively; *p* = 0.034) [91]. Elevated distress levels were associated with the resumption of daily practice after the lockdown period, and 31.2% of orthodontists with higher distress were in favor of interrupting their work activity. Their main fear was the possibility of infecting a family member, which was greater than the concerns about their own death (48.2% vs. 26.9%, respectively) [51].

Finally, some of the studies comparing the impact of the pandemic on dental professionals with respect to other healthcare groups (as physicians or nurses) showed a higher prevalence of anxiety symptoms in dentists, and a greater reduction in their work activity [25,33]. On the other hand, it has also been described that financial uncertainty appears to have negatively influenced the emotional state of dental practitioners [40,53,65,78]. The evaluation over time of this financial insecurity and its impact on professionals was not analysed in any study.

### 3.4. Impact of COVID-19 on Patients

Twenty-four studies focused on patients receiving general dental treatment [76,90,95,98,100,108,109,110,111,112,113,114,115,117,119,120,123,124,126,129,130,132,133,134], ten on patients under orthodontic treatment [96,102,104,105,116,118,125,128,136,137], six on pediatric patients [97,99,101,103,107,122], two on patients with temporomandibular disorders [106,135], one on patients under endodontic treatment [121], one on prosthodontic patients [131], and one on patients undergoing oral surgery [127].

Among adult patients receiving general dental treatment (restoration, extraction, cleaning, etc.), the main study variable analyzed was the fear of visiting the dentist during the pandemic (n = 11), reaching up to 63.6% of these patients [115]. Various studies [95,100,110,111,112,113,115] found a greater reluctance to seek dental treatment and a more marked tendency to postpone appointments among female patients and among over 60-year-olds who had a systemic disease. The delay in dental care, mainly due to restrictive measures, was associated with depression in adults of middle age (Odds Ratio (OR): 2.05, 95% confidence interval (CI): 1.04–4.03) and in those over 65 years old (OR: 3.08, 95% CI: 1.07–8.87) [114].

Most orthodontic patients appeared willing to continue their treatment, with 69% reporting that the sole reason for its interruption was the closing of their dental clinic [102]. A possible delay in their treatment was found to be the main concern of these patients [105,118,137]. Anxiety was described in almost half of them, observing that females were more prone to suffer both anxiety (5.35 ± 2.48 vs. 4.29 ± 2.18 in males on a 10-point scale; *p* < 0.001) [105] and psychological distress (OR: 1.77, 95% CI: 1.07–2.93) [136].

Studies in the pediatric setting confirmed that parents were less willing to take their children to the dentists when their fear of COVID-19 infection was greater, and 66% of parents only sought dental care when emergency treatment was required [103]. A higher level of anxiety about visiting the dentist was shown by children during the pandemic, although it was not significantly greater than pre-pandemic levels evaluated in 2018, and their anxiety was lesser with older age. Anxiety levels in caregivers were also higher than those observed in 2018 and were more strongly correlated (close to 1) with the anxiety of the children [122].

## 4. Discussion

This review evidences the high levels of anxiety experienced by dental professionals during the COVID-19 pandemic, similarly to the findings of previous reviews [10,11], mainly caused by fears of infection and of work insecurity. Professionals who were younger and female appeared more vulnerable to these concerns. Fears raised by the pandemic also had a psychological impact on patients, leading them to avoid or postpone dental treatments.

As it has been described previously, it was found a greater reduction in the work activity of dentists in comparison to physicians and nurses, among others [25,33]. The relationship of dentists with patients and colleagues was also more strongly affected by the pandemic, attributed to a lesser feeling of safety and preparation for the treatment of possibly infected patients [33]. Although dentists and physicians both expressed major worries about the risk of infecting their family members, these appeared to be greater among dental professionals [33]. In this regard, it has been observed that work-related stress levels are higher among dentists than among other healthcare professionals under “normal” (non-pandemic) conditions [139]. In fact, studies have suggested that dentists are more prone to professional burnout, anxiety and depression, even when they are still dental students [140,141,142,143]. Nevertheless, these studies showed a maximum prevalence of both variables that did not reach 45%, while a large number of studies included in this review far exceed that percentage, presumably as a consequence of the COVID-19 pandemic.

Dental professionals frequently described a lack of agreement on the effectiveness of available preventive measures (e.g., air purifiers, ozone generators, etc.), exacerbating the psychological problems observed. In this context, a key factor during the early phase of the pandemic was the difficulty obtaining personal protection equipment or material (e.g., surgical masks, safety glasses, face shields, etc.) [144]. Some professionals even asked acquaintances with 3D printers to manufacture protective shields [145]. The insecurity generated by this shortage was one of the most frequent complaints cited by professionals in the reviewed studies.

Consistent with previously described results [11], the financial insecurity generated by the pandemic also appears to have influenced the emotional state of dentists. Dental offices had to adapt to the new situation, investing heavily in all types of protective measures for clinicians and patients. Being an eminently private profession, this added expense may have further impacted on the psychological stability of dentists. Furthermore, although there was a smaller volume of patients than before the pandemic, protective measures inevitably increased the time devoted to each one. The correct disinfection and ventilation of the dental office, completion of an exhaustive questionnaire to identify possible symptoms, and other associated administrative tasks were responsible for work overload in all members of the clinical team, with a higher risk of burnout syndrome [40,54,63,67,79]. Moreover, increased anxiety, stress and burnout were detected among dental professionals working in hospitals [18,29,50,63], not because of an increase in workload during the pandemic due to the reduced private clinical activity [8] but rather attributable to greater contact with possible carriers of SARS-CoV-2 in the hospital setting [146,147].

The studies reporting significant differences in psychological disorders as a function of sex or age were of high methodological quality and indicated that anxiety and depression were more frequent among the younger age groups, similar to the finding of a previous review [11], and female dentists. One explanation may be that younger professionals tend to have a higher workload and to be less financially stable, as previously observed in other types of healthcare professionals [148]. Regarding the apparently greater effect on female professionals, it should be born in mind that females are considered two-fold more likely to suffer from anxiety than males in the general population [149]. Fears about the possibility of virus transmission to their children may also have been greater in dentists who were mothers [50].

Many patients perceived the dental office as an unsafe environment and expressed high levels of fear about possible infection in the waiting room or during treatment [95,104,111,115,133]. As with all diseases, a delay in dental treatment can have negative health consequences [150,151], and the increased consumption of sugar observed during the pandemic [119,130] would further contribute to a worsening of oral and general health. In this regard, it has been suggested that the presence of periodontal disease in patients diagnosed with COVID-19 may be related to higher complication and mortality rates [152]. It is essential to warn populations about the negative effects of postponing dental treatments due to COVID-19 and to assure them that the dental office is a safe setting.

Unlike general dental care, most patients receiving orthodontic therapy wanted it to be continued without interruption. Although the treatment is non-invasive, aerosols can be generated by some procedures such as debonding [153]; however, patients may be unwilling to disrupt a long-term orthodontics plan that has already been started. It is also possible that patients establish a more direct relationship with their orthodontists, especially via mobile apps; in fact, the marked increase in “teledentistry” during the pandemic has demonstrated its usefulness to enhance the relationship between patient and healthcare professional [154].

A visit to the dentist frequently produces anxiety in children [155], and high anxiety levels have been strongly correlated with those of their parents, known to play a key role in the potential development of anxiety disorders in their children [156]. At the beginning of the pandemic, little was known about COVID-19 impact on children and reports were controversial, ranging from the possibility to develop Kawasaki syndrome [157] to assurance that symptoms were mild in children, with a good prognosis [158]. This uncertainty may have increased the reluctance of parents to seek non-emergency dental care for their children. One way to reduce anxiety is for professionals to convince patients that it is safe to come to the office and receive treatment [122].

Despite data collection occurring at different pandemic moments (high and low incidences of COVID-19 depending on the study), it is unlikely that this had impact on the psychological impact since the vaccination process had not been initiated in the majority of them and the fear due to this lack of protection may be generalized for all studies included in this review [159]. Indeed, in one of the few studies that included vaccinated healthcare professionals, receipt of vaccination was associated with a reduction of fear and anxiety levels in 35.6% of participants [160]. Vaccinated patients may also be more willing to seek treatment, and Vohra et al. [133] reported that 62.4% of patients were ready to receive treatment after their vaccination.

Of note, the percentage of subjects with anxiety and fear was usually higher in those studies that were performed with non-validated questionnaires. This is relevant regarding the confidence in the results obtained by means of non-validated tools as they may be subject to measurement error, as it has been suggested in other areas [161].

There is a need for longitudinal studies to determine the persistence of these psychological effects of the pandemic and to investigate possible associated factors. Data obtained could assist to the development of psychological support protocols that allow healthcare professionals to carry out early interventions to prevent the worsening of anxiety, depression, or stress.

Limitations of this review include that the comparisons of results among studies are hampered by their utilization of distinct instruments and methodologies to evaluate each psychological aspect, although most studies used validated scales relatively frequently applied in the field of psychology. It would be advantageous to unify criteria in future investigations, prioritizing instruments that specifically evaluate the relationship between COVID-19 and its possible emotional consequences, such as the “Fear of COVID-19 Scale, FCV-19S” [162] and “COVID-19 Peri-Traumatic Distress Index, CPDI” [163] described in this review. Quantification of the results obtained was also limited by the heterogeneity of the analytical methodologies applied. In addition, the data on which studies were based were self-reported by the individuals and therefore subjective, potentially differing from a potential professional psychological diagnosis. Finally, most of the evidence found was based on cross-sectional studies, with only seven having a longitudinal design, preventing confirmation that the psychological/emotional disorders observed were caused by the pandemic.

One strength of this review is that it is the first to jointly consider the emotional impact of the pandemic on professionals and patients, offering a more global view of clinical dentistry. The external validity of the review may be supported by the fact that the studies were conducted in numerous different countries and did not focus on a specific epidemiological area or context, obtaining a methodological quality score > 4 stars in 96%. Finally, no article was excluded due to its language, despite being an inclusion criteria, which may imply a lower selection bias.

## 5. Conclusions

The COVID-19 pandemic had a major impact on dental practice, raising the anxiety levels of the professionals, increasing the patients’ fear of visiting their dentist, and being responsible for multiple psychological disorders in both groups. Further studies are needed to evaluate the possible persistence of these disorders over time and once the vaccination process has been widely established.

## Figures and Tables

**Table 1:** Characteristics of studies on dental professionals using validated scales.

First Author/Publication Year Quality Assessment	Study Location	Sample Size	Field of Practice	Characteristics and Period of Data Collection	Variable [Source of Information]	Main Results
Ajwa, N. et al./2020 [16] NOS: 8/10	Saudi Arabia	577	179 Dentists 46 Dental assistants 6 Dental hygienists 346 Other healthcare combinations	Age not reported 49% Female From June to August 2020	(1) Anxiety [GAD-7] (2) Depression [PHQ-9]	18% of dentists had moderate anxiety, and 6% showed severe symptoms. Regarding depression, 33% reported moderate to severe levels, with only 5% showing severe symptoms. Neither the dental assistants nor the dental hygienists reported such severity.
Aldhuwayhi S. et al./2021 [17] NOS: 6/10	Saudi Arabia	206	Dentists (unspecified)	31–40 years (44.2%) 30.6% Female 2020	(1) Perceived stress [PSS-10] (2) Stress busters and coping mechanisms [Non-validated questionnaire]	Male dentists, those ≥50 years of age, and private practitioners obtained higher stress scores. The main stress busters were using technology (80%), binge eating (64%), exercise (44%) and smoking (32%).
Alencar C. de M. et al./2021 [18] NOS: 8/10	Brazil	998	Dentists (unspecified)	39.39 years ±11.69 72.7% Female From 11 to 30 July 2020	Depression, anxiety and stress [DASS-21]	Dentists living with someone at high risk for COVID-19, who did not practice leisure activities, working on the frontline, and those who suffered changes in their eating habits, sleep quality and physical health during this period obtained the highest scores for anxiety, depression and stress.
Bellini P. et al./2020 [24] NOS: 5/10	Italy	1109	Dentists (unspecified)	35–55 years (44.3%) 29.6% Female From 2 to 29 April 2020	Anxiety [GAD-7]	13.9% of dentists reported moderate anxiety, while 6.4% showed a severe level. No significant differences were found when comparing two groups based on the number of cases registered in their work area (more/less than 15,000 confirmed cases).
Campos J.A.D.B. et al./2021 [25] NOS: 6/10	Brazil	1609	341 Dentists 1268 Other healthcare professionals	36.9 years ±11.6 83.2% Female 16.3% Male 0.5% Other From 18 May to 13 June 2020	(1) Depression, anxiety and stress [DASS-21] (2) Posttraumatic stress [IES-R]	Dentists had the highest out-of-work prevalence (32.3%) and the lowest prevalence of remote work (20.2%) among healthcare professionals. Regarding the psychological variables, the prevalence of depression, anxiety and stress symptoms was higher in dentists than in physicians, nurses and psychologists.
Chakraborty T. et al./2020 [27] NOS: 7/10	India	335	168 Students 167 Dentists	Students: 24 years ±3 82% Female Dentists: 31 years ±4 63% Female From 1 to 10 May 2020	Depression [PHQ-9]	44% of dentists showed moderate to severe levels of depression, with higher prevalence in males, younger than 30 years old, and those with fear of contracting COVID-19 from the patients.
Chaudhary F.A. et al./2021 [28] NOS: 7/10	Pakistan	392	254 Dentists 138 Dental assistants/hygienists	20–39 years (62.2%) 54.8% Female From April to July 2020	(1) Anxiety [GAD-7] (2) Posttraumatic stress [IES-R]	25% of professionals reported moderate to severe anxiety levels, and only 14% had post-traumatic stress disorder. About 26% were willing to treat patients, and higher anxiety and stress levels were associated with females, older age, and living with the close ones.
Chen Y.; Li W./2021 [29] NOS: 9/10	China	808	558 Dentists 250 Nurses	36.20 years ±8.21 30.9% Female From 3 to 10 April 2020	(1) Anxiety [GAD-7] (2) Depression [PHQ-9] (3) Perceived stress [PSS-10] (4) Posttraumatic stress [ASDS]	The prevalence of anxiety, depression, perceived stress, and post-traumatic stress was 36.3%, 46.4%, 65.2%, and 1.1%, respectively. Frontline dental professionals working in the Wuhan area and those with a past medical history reported higher anxiety and perceived stress.
Collin V. et al./2021 [31] NOS: 6/10	United Kingdom	5170	4384 General dentists 786 Other	40–49 years (28.9%) 49% Female 50.3% Male 0.7% Other From 22 to 27 May 2020	Psychological distress [GP-CORE]	The highest levels of psychological distress among UK dental professionals were observed in those from England, but it was lower in UK dentists during the national lockdown period when compared to a previous research conducted in 2018 using the same questionnaire.
Consolo U. et al./2020 [32] NOS: 8/10	Italy	356	Dentists (unspecified)	35–55 years (48.6%) 39.6% Female From 2 to 21 April 2020	Anxiety [GAD-7]	9% of dentists showed severe anxiety levels. 85.2% were concerned about getting COVID-19 during clinical practice, and 89.6% reported being worried about their professional future.
Dreher A. et al./2021 [35] NOS: 7/10	Germany	1481	Dental assistants	35 years (IQR: 28–42) 98.4% Female From 7 to 14 April 2020	(1) Anxiety [GAD-7] (2) Depression [PHQ-2]	The prevalence of anxious and depressive symptoms was 48.8% and 39.2%, respectively. Main stressors referred were uncertainty about the temporal scope of the pandemic (97.9%), financial situation (87.8%) or thoughts about a possible infection during clinical activity (83.8%).
Estrich C.G. et al./2021 [36] NOS: 7/10	United States	4776	Dental hygienists	44.1 years ±12.0 98.1% Female 1% Male 0.9% Other From September 29 to October 8 2020	(1) Anxiety and depression [PHQ-4] (2) Concern about COVID-19 transmission to patients and to themselves [Validated with a pilot study]	Dental hygienists showed elevated symptoms of anxiety (25.7%) and depression (16.05%), being both significantly associated with the 18–29 age group. 3.1% had been diagnosed with COVID-19, and 99.1% reported enhanced infection control efforts.
Ortega-López M.F. et al./2021 [38] NOS: 7/10	14 Latin American countries Most from Peru (23.7%), Ecuador (21.6%), Venezuela (12.2%) and Brazil (8.6%)	139	Pediatric dentists	41.16 years ±10.88 94.2% Female 2020	(1) Perceived stress [PSS-14] (2) Subjective well-being [Subjective Well-being Scale of the WHO]	Perceived stress decreased as the age of dentists increased one year. Similarly, increasing age was related to a decrease in the perception of affective support, and professionals in mandatory quarantine reported a greater feeling of not having emotional support.
Gasparro R. et al./2020 [39] NOS: 7/10	Italy	735	Dentists (unspecified)	44.8 years ±12.44 32.7% Female From 17 April to 3 May 2020	(1) Fear of COVID-19 [FCV-19S] (2) Depression [SMDA]	Both fear of COVID-19 and perceived job insecurity were positively associated with depressive symptoms. Among those dentists who showed low fear levels of COVID-19, the effect of perceived job insecurity on these symptoms was weaker.
Humphris G. et al./2021 [40] NOS: 8/10	United Kingdom (Scotland)	328	110 Dental trainee 218 Dentists in primary dental care	Dental trainee: 26 years ±5 79.1% Female Dentists in primary dental care: 43 years ±11 81.2% Female 19.3% Male 0.5% Other From August to October 2020	(1) Emotional exhaustion [Non-validated questionnaire] (2) Burnout [MBI] (3) Depression [PHQ-2] (4) Posttraumatic stress [IES-R]	Around 27% of all participants reported significant depressive symptoms, and 55% of primary care staff were emotionally exhausted. In addition, they felt less prepared for managing their health, and coping with financial uncertainty and insecurity when compared with the trainees.
Iorga M. et al./2021 [41] NOS: 6/10	Romania	83	38 General dentists 19 Consultants 16 Residents 10 Specialists	37.81 years ±8.45 63.9% Female 34.9% Male 1.2% Other From 18 November to 5 December 2020	(1) Fear of COVID-19 [FCV-19S] (2) Insomnia [Athens Insomnia Scale]	There was a strong positive correlation between the total scores of insomnia and fear of COVID-19. The more the professionals were afraid of patients lying about their health, or getting infected from their co-workers or while taking off their personal protective equipment, the more severe their insomnia was.
Kale P. et al./2021 [42] NOS: 7/10	India	600	Dentists (unspecified)	30.93 years ±8.71 67.8% Female From September to October 2020	Anxiety [GAD-7]	37% of dentists showed mild signs of anxiety; about 40% had moderate anxiety, and 23% of professionals displayed severe signs of anxiety.
Kamal A.T. et al./2021 [43] NOS: 9/10	Pakistan	85	25 General dentists 36 Specialists 24 Dental assistants	31.6 years ±6.0 62.4% Female From 20 July to 5 August 2020	(1) Perceived stress [PSS] (2) Anxiety [GAD-7]	The main reasons for stress and anxiety were fear of getting infected (91.8%), the possibility of transmission to the family (87.1%), and aerosol-generating procedures (84.7%). Female gender and age were significantly associated with higher scores for both variables.
Kirli M.C.; Kirli U./2021 * [48] NOS: 6/10	Turkey	81	14 Endodontists/Restorative dentistry 17 Nurses 19 Cleaning/Data entry staff 31 Other departments	30.3 years ±6.8 50.6% Female 2020: Before and after restarting high-risk procedures	Anxiety [STAI]	It was observed that the anxiety level of professionals increased significantly on the day that the high-risk procedures were restarted. This increase was significant for females, dentists working in endodontics and restorative dental care, and nurses.
Labban N. et al./2021 [49] NOS: 8/10	Saudi Arabia	202	Dentists (unspecified)	21–30 years (49.5%) 71.3% Female From July to August 2020	(1) Concerns about treating patients and state of worry [Validated with a pilot study ](2) Anxiety [GAD-7] (3) Posttraumatic growing [PTGI-SF]	63.4% of dentists had a score greater than 40 on the anxiety scale from 0 (no anxiety) to 100 (highest anxiety). 44.1% were willing to continue treating their patients during this period, and more than 50% worried about not being able to do it in the personal way as before.
Martina S. et al./2020 [51] NOS: 7/10	Italy	349	Orthodontists	30–39 years (31.5%) 49.9% Female From 1 to 6 May 2020	(1) Perceived risk, anxiety, distress and fears for an infection [Non-validated questionnaire] (2) Anxiety and depression [PHQ-4]	22% of professionals showed a moderate/high level of distress, and 31.2% of them were inclined to interrupt their activity. For 55.2% of dentists, returning to their daily practice was a source of anxiety, which was significantly associated with the level of distress.
Mekhemar M. et al./2021 [52] NOS: 9/10	Germany	252	Dental nurses	18–49 years (76.5%) 98% Female From July 2020 to January 2021	(1) Depression, anxiety and stress [DASS-21] (2) Posttraumatic stress [IES-R]	The percentages of participants with moderate to extremely severe depression, anxiety and stress were 31%, 25.3% and 28.6%, respectively. Having immune-deficiency or chronic diseases, working in a dental practice, and perceiving the pandemic as a financial threat were revealed as significant risk factors with higher scores.
Mekhemar M. et al./2021 [53] NOS: 9/10	Germany	732	Dentists (unspecified)	18–49 years (53.3%) 59.7% Female 40% Male 0.3% Other From July to November 2020	(1) Depression, anxiety and stress [DASS-21] (2) Posttraumatic stress [IES-R]	The percentages of participants with moderate to extremely severe depression, anxiety and stress were 28.9%, 18.2% and 29.6%, respectively. Higher scores on both assessing scales were significantly associated with female gender, 50–59 age group, being immune-deficient or chronically ill, working in a dental practice, and considering the pandemic a financial risk.
Mijiritsky E. et al./2020 [54] NOS: 7/10	China, India, Israel, Italy & United Kingdom	1302	Dentists (unspecified)	34.9 years ±9.4 to 47 years ±11.4 (depending on the country) 41.2% Female From 30 March to 12 April 2020	(1) Fear of contracting COVID-19 from patients/family [Non-validated questionnaire] (2) Psychological factors [Demands Scale] (3) Psychological distress [Kessler’s K6]	The positive association between subjective overload and psychological distress suggested a higher rate of intensity in Italy, when compared to the rest of the countries. The interaction variable between both of them was significantly associated with the UK and with those dentists who reported fear of contracting COVID-19 from patients, or their families becoming infected.
Mishra S. et al./2020 * [55] NOS: 8/10	India	1253	Dental practitioners & Academicians	33.28 years ±7.64 51.55% Female Phase I: from 20 to 25 March 2020 Phase II: from 25 to 30 April 2020	(1) Perceived stress [PSS] (2) Sources of stress [Non-validated questionnaire]	Perceived stress increased by more than two points (on a scale of 40) from phase I (before the onset of SARS-CoV-2 spread) to phase II (in the month immediately following the nationwide lockdown) of the pandemic. Lack of family time due to long working hours was the main stressor (90%) among professionals during phase I, and concern about getting infected was the most frequent (83.3%) during phase II.
Mulla S. et al./2020 [57] NOS: 6/10	India	126	Dentists (unspecified)	23–30 years (77.6%) 54.8% Female From 25 March to 25 May 2020	Fear of COVID-19 [FCV-19S]	Dentists were afraid due to the effects of the pandemic. 96.03% reported apprehension about patient safety from COVID-19, and females showed less confidence in living with the virus.
Nagarajappa R. et al./2021 [58] NOS: 7/10	India	234	Dentists (unspecified)	30.58 years ±6.70 70.1% Female From June to August 2020	Psychological distress [CPDI]	There was a statistically significant association of higher scores of psychological distress with age, gender, practice and education. The odds of stress were two times higher among males.
Nair A.K.R. et al./2020 [59] NOS: 7/10	India	586	Endodontists	25–35 years (55%) 46.9% Female From 8 to 16 April 2020	(1) Perceived stress [PSS] (2) Psychological distress [CPDI]	4 out of 5 endodontists were stressed, with females showing a higher level. 1 out of 2 endodontists had distress, being higher in the ≤35-year-old group, compared to ≥45-year-old group.
Olivieri J.G. et al./2021 * [61] NOS: 7/10	Spain	15	4 Endodontists 11 Dental assistants	29–36 years Endodontists (Not reported in Dental assistants) Gender not reported Phase I: from 27 March to 21 May 2020 Phase II: from 26 May to 18 June 2020	(1) Anxiety [GAD-7] (2) Perceived anxiety, stress and safety [Non-validated questionnaire]	General anxiety decreased over the weeks, with significant differences between strict and partial confinement. Endodontists showed higher levels of anxiety during anesthesia inoculation, and dental assistants during dental unit’s disinfection.
Özarslan M.; Caliskan S./2021 [63] NOS: 8/10	Turkey	706	330 Dentists serving in the filiation service 376 Dentists not serving in the filiation service	22–30 years (56.1%) 76.5% Female Not reported in the second stage of the study From 9 March to 20 May 2020	(1) Stress [Validated with a pilot study] (2) Burnout [MBI]	Most dental professionals showed greater levels of stress, being significantly higher in those working in the filiation service (identification and management of possible COVID-19 cases). In addition, 34.4% of them reported occupational burnout, compared to 17.6% of those not working in that service.
Peixoto K.O. et al./2021 [66] NOS: 7/10	Brazil	641	Dentists (unspecified)	39 years ±10.56 74.1% Female 25.4% Male 0.5% Other From May to June 2020	(1) Depression, anxiety and stress [DASS-21] (2) Sleep quality [PSQI]	Depressive symptoms were significantly higher in quarantined dentists. Less worry about the pandemic was associated with less odds of experiencing stress, anxiety, and poor sleep quality. Sleep showed a strong positive correlation with psychological factors in frontline workers.
Ranka M.S.; Ranka S.R./2021 [71] NOS: 7/10	United Kingdom	123	Dentists (unspecified)	Not reported June 2020	(1) Anxiety and depression [PHQ-4] (2) Stress [NRS]	The prevalence of anxiety, depression and stress corresponded to 71%, 60% and 92%, respectively. Dentists working in the private sector showed more psychological symptoms compared with those in the public sector.
Salehiniya H.; Abbaszadeh H./2021 [72] NOS: 7/10	Iran	320	232 General dentists 88 Specialists	44.38 years ±10.66 46.25% Female From 2 to 14 May 2020	(1) Anxiety [Professional validation] (2) General health [GHQ-28]	42.5% of professionals showed anxious symptoms, with 32.5% out of them reporting mild anxiety. 35% of professionals had mild psychiatric disorders, and there was a significant relationship between history of physical illness and psychiatric disorders with COVID-19 associated anxiety.
Sarapultseva M.et al./2021 [74] NOS: 6/10	Russia	128	43 General dentists 48 Dental auxiliaries 37 Dental assistants	38.6 years ±13.9 78.9% Female From 1 to 20 September 2020	(1) Depression, anxiety and stress [DASS-21] (2) Psychological distress [IES-R] (3) Posttraumatic stress [PSS-SR]	Around 22% of professionals had mild to extremely severe psychological distress symptoms, and up to 29.7% showed clinical symptoms of post-traumatic stress, with significantly higher levels in older workers for both variables. Scores for DASS-21 were normal in almost 80%.
Serota K.S. et al./2021 [78] NOS: 7/10	Hungary	182	Dentists (unspecified)	50.93 years ±13.67 65.9% Female 2021	(1) Perceived stress [PSS] (2) Psychological distress [Non-validated questionnaire] (3) Concerns about the COVID-19 pandemic [Non-validated questionnaire]	Dentists reported a lack of interest in social relationships, mood swings and emotional exhaustion. Fear of aerosol propagation and financial insecurity increased the probability of higher levels of perceived stress and distress, while years of practice and age seemed to be protective factors.
Shacham M. et al./2021 [79] NOS: 7/10	Israel	338	198 Dentists 140 Dental hygienists	46.39 years ±11.18 58.6% Female From 30 March to 10 April 2020	(1) Fear of contracting COVID-19 from patients/family contracting [Non-validated questionnaire] (2) Psychological factors [Demands Scale] (3) Psychological distress [Kessler’s K6]	Greater psychological distress was found among professionals who had a background illness, fear of getting COVID-19 from the patient, and a higher subjective overload. Lower psychological distress was associated with being in a committed relationship and greater self-efficacy scores.
Tao J. et al./2021 [83] NOS: 9/10	China	969	Dentists (unspecified)	35.55 years ±8.26 68% Female From 3 to 10 April 2020	(1) Depression [PHQ-9] (2) Anxiety [GAD-7] (3) Perceived stress [PSS-10] (4) Posttraumatic stress [ASDS]	66.3% of participants reported more than one psychological symptom, with perceived stress being the most prevalent (66.2%) and anxiety the least (7.1%). Dentists with preexisting physical health conditions had increased risk of depression and perceived stress.
Uziel N. et al./2021 [89] NOS: 6/10	Israel, Canada & France	537	302 Dental practitioners 235 Para-dental personnel	Dental practitioners: ±45–50 years 50% Female Para-dental personnel: 40 years approx. 100% Female From 18 April to 13 June 2020	(1) Anxiety and depression [PHQ-4] (2) Attitudes towards patients [Non-validated questionnaire] (3) Posttraumatic growing [PTGI-SF]	Israeli professionals were less concerned about their physical and mental health, and their social relationships. Canadians were most willing to treat their patients, and most worried about not being able to do so as before the lockdown. French professionals reported the highest level of fear to treat patients.
Yilmaz H.N.; Ozbilen E.O./2020 [91] NOS: 6/10	Turkey	215	Orthodontists	20–34 years (52.1%) 70.2% Female From 6 to 15 June 2020	Anxiety [GAD-7]	16.7% of orthodontists were anxious, with a statistically significant difference between the working place and the level of anxiety. Thus, the odds of having anxiety above the threshold were higher among those working in public institutions and organizations (60%).
Yılmaz M. et al./2021 [92] NOS: 8/10	Turkey	434	52 Dentists 278 Physicians 104 Nurses	<35 years (70.7%) 65% Female From 1 April to 1 May 2020	(1) Sleep quality [PSQI] (2) Related factors to sleep quality: * Perceived social support [MSPSS] * Posttraumatic stress [NSESSS]	The prevalence of poor sleep quality was 42.3% in dentists, the lowest as compared to doctors (55.4%) and nurses (67.3%). High levels of social and family support were identified as protective factors, and poor sleep quality was significantly associated with working in hospitals and high post-traumatic stress levels.
Zeidi I.M.; Zeidi B.M./2021 [93] NOS: 8/10	Iran	340	246 General dentists 35 Oral surgeons 30 Orthodontists 11 Periodontists 8 Pediatric dentists	37.54 years ±11.50 46.8% Female From 4 April to 18 July 2020	Fear of COVID-19 [COVID-19 Fear Questionnaire]	82.1% of professionals were afraid of getting infected by patients, treating to the suspected ones, transmitting the infection to their relatives, post-infection quarantine and treatment costs. Job history, knowledge, attitude, and fear were significant predictors of dentists’ practice.
Zhao S. et al./2020 [94] NOS: 7/10	China	269 Professionals 258 General public	Frontline dental staff	Professionals: 27.2 years ±8.4 68.4% Female General public: 40.53 years ±10 61.24% Female From 2 to 13 May 2020	Professionals: (1) Anxiety [Beck Anxiety Inventory] (2) Potential factors associated with the anxiety [Non-validated questionnaire]: * Working conditions * Protective measures General public: Anxiety [Beck Anxiety Inventory]	Frontline dental professionals were 4.3 times more likely to suffer anxiety than the general public. An elder age and level 3 protection measures would decrease their anxious level, whereas the conflict with patients or colleagues could worsen it.

ASDS: Acute Stress Disorder Scale; CPDI: COVID-19 Peritraumatic Distress Index; DASS-21: Depression, Anxiety and Stress Scales-21; IES-R: Impact of Event Scale-Revised; GAD-7: Generalized Anxiety Disorder-7; GHQ-28: General Health Questionnaire-28; GP-CORE: General Population-Clinical Outcomes in Routine Evaluation; FCV-19S: Fear of COVID-19 Scale; IES-R: Impact of Event Scale-Revised; MBI: Maslach Burnout Inventory; NRS: Numeric Rating Scale; MSPSS: Multidimensional Scale of Perceived Social Support; NSESSS: National Stressful Events Survey PTSD Short Scale; PHQ-2/-4: Patient Health Questionnaire-2/-4; PHQ-9: Patient Health Questionnaire-9; PSS-10: Perceived Stress Scale-10; PSS: Perceived Stress Scale; PSS-14: Perceived Stress Scale-14; PSS-SR: Post-traumatic stress disorder Symptom Scale-Self Report; PSQI: Pittsburgh Sleep Quality Index; PTGI-SF: Post-Traumatic Growth Inventory-Short Form; SMDA: Severity Measure for Depression-Adult; STAI: State-Trait Anxiety Inventory. All of the studies had a cross-sectional design, except those marked with *.

**Table 2:** Characteristics of studies on dental professionals using non-validated scales.

First Author/Publication Year Quality Assessment	Study Location	Sample Size	Field of Practice	Characteristics and Period of Data Collection	Variable [Source of Information]	Main Results
Ahmed M.A. et al./2020 [15] NOS: 6/10	30 countries worldwide Most from Pakistan (30.8%)	650	511 General dentists 97 Specialists 42 Consultants	20–40 years (92.84%) 75% Female From 10 to 17 March 2020	Fear and anxiety [Non-validated questionnaires]	78% of the general dentists were scared and anxious by the effects of COVID-19. 66% of dentists preferred to interrupt their practice until the number of active cases declined. 76% worked in the hospital setting, out of which 74% were in private hospitals.
Alkhalifah F.N. et al./2021 [19] NOS: 5/10	Saudi Arabia	118	Dental hygienists	20–30 years (67.8%) 73.7% Female From 15 to 28 May 2020	Stress and well-being [Professional validation]	73.7% of dental hygienists had not provided care or treatment during quarantine, and 65.3% reported moderate stress level regarding going back to work. 22.9% admitted having been forced to work under conditions that could jeopardize their personal safety.
Aly M.M.; Elchaghaby M.A./2020 [20] NOS: 6/10	Egypt	216	113 General dentists 86 Specialists 17 Consultants	20–40 years (84.2%) 44% Female 2020	Fear of being infected [Non-validated questionnaire]	92.6% of dental professionals were afraid of being infected with SARS-CoV-2, while 90.7% were anxious about treating patients showing suspicious symptoms of COVID-19.
Amato A. et al./2021 [21] NOS: 6/10	Italy	849	Dentists (unspecified)	>50 years (46.1%) 30.7% Female From 26 April to 3 May 2020	Attitudes toward the COVID-19 infection [Non-validated questionnaire]	88.3% of dentists were worried about their relatives’ health, and 73.9% were also concerned about a possible infection among their collaborators. About 86% of dentists reported some income loss, and 94% were afraid of a decrease in the number of patients after the quarantine.
Aurlene N. et al./2021 [22] NOS: 6/10	India	32	Dentists (unspecified)	Age not reported 59.4% Female From June to September 2020	Challenges faced in dental practice and social life impact [Focus group discussions]	The main challenges faced in dental practice were the confusion in COVID-19 protocols, concerns over growing costs, fear of being infected and transmitting to family members, negligent patient attitudes, and the uses and limitations of teledentistry.
Balkaran R. et al./2021 [23] NOS: 5/10	10 Caribbean countries Most from Trinidad and Tobago (77.6%)	152	129 General dentists 23 Specialists	35–45 years (38.8%) 58.6% Female From December 2020 to March 2021	Fear of being infected [Validated with a pilot study]	75% of general dentists were stressed, with 80.9% reporting financial impact. 94.7% believed that aerosol-generating procedures involve the highest risk of COVID-19 transmission, and 87.5% were worried about contracting it clinically. 69.1% were willing to receive the vaccine.
Çelik O.E.; Cansever İ.H./2021 [26] NOS: 6/10	Turkey	734	Dentists (unspecified)	20–40 years (55.4%) 54.4% Female From 30 September to 20 October 2020	Anxiety and stress [Non-validated questionnaire]	80.8% of dentists were anxious about examining patients during the pandemic. Anxiety levels increased with increasing the number of patients seen per day, and decreased with increasing the dentist’s age. 85.8% were worried about their professional future, especially in governmental practices.
Cheng H-C. et al./2021 * [30] NOS: 6/10	Taiwan	276 (2018) 251 (2020)	Dentists (unspecified)	May 2018 >50 years (64.91%) 19.2% Female April 2020 >50 years (64.97%) 17.1% Female	Fear of being infected [Professional validation]	94% of dentists were afraid of becoming infected with SARS-CoV-2. Around 95% wore personal protective equipment, with a significant increase in the number of those wearing hair caps and face shields when compared to the pre-pandemic period.
Cotrin P. et al./2020 [33] NOS: 7/10	Brazil	536	187 Dentists 179 Physicians 170 Nurses	31–40 years (44.1%) Dentists: 66.8% Female Physicians: 65.4% Female Nurses: 88.8% Female 2020	Fear and anxiety [Non-validated questionnaire]	91% of dentists reported being afraid of becoming infected in the clinical environment, and 98% changed habits due to fear of infecting their relatives. Dentists were more anxious than physicians, and their relationship with patients was also more influenced by the pandemic.
De Stefani A. et al./2020 [34] NOS: 5/10	Italy	1500	243 Orthodontists 1257 Other combinations	30–49 years Female (61.2%) ≥40 years Male (69%) 55.7% Female From 11 to 18 April 2020	Attitude in treating potentially infected patients [Non-validated questionnaire]	65.7% of professionals would have refused to treat a patient suffering from a runny nose and cough. Only in a dental emergency, some would have treated them, wearing personal protective equipment (8.2%) or by prescribing the PCR test (8.2%) after treatment. 9.6% would have referred the patient to the National Healthcare Service.
Fairozekhan A.T. et al./2021 [37] NOS: 6/10	7 Asian countries Most from India (47%)	788	566 Dental professionals 222 Other healthcare professionals	<35 years (59.5%) 55.5% Female From 15 April to 5 May 2020	Perceived stress and job-related concerns [Validated with a pilot study]	Dental professionals were stressed due to the pandemic situation, although significantly less than other medical professionals regarding seeing co-workers displaying symptoms, as well as the possibility of transmitting COVID-19 to their family or friends.
Kamran R. et al./2021 [44] NOS: 5/10	Pakistan	313	107 General dentists 72 Consultants/Specialists 134 Other	20–40 years (83%) 57.2% Female From 16 to 20 June 2020	Fear and anxiety [Professional validation]	75% of professionals were afraid of becoming infected, and 92% were concerned about transmitting the virus to their relatives. 88% were anxious when treating suspected COVID-19 patients, although only 28% were using rubber dam isolation, and 68% avoided aerosol-generating procedures.
Karayürek F. et al./2021 [45] NOS: 5/10	Turkey	947	607 General dentists 340 Specialists	31.72 years 62% Female 2020	Fear and anxiety [Non-validated questionnaire]	Most professionals showed an increase in fear and anxiety levels: 74.9% were working in universities, followed by 68.1% professionals in public institutions, and 64.6% working in private clinics. Those under 30 years of age presented significantly higher rates for both variables.
Karobari M.I. et al./2021 [46] NOS: 6/10	India, Malaysia, Saudi Arabia, Pakistan, United Kingdom & Cambodia	272	162 General dentists 110 Specialists	Age not reported 54% Female 44.9% Male 1.1% Other From February to May 2021	Stress and smoking-related behavior [Validated with a pilot study]	15% of dental professionals reported started smoking or increasing their smoking habit during the pandemic because of stress. 6.8% were not sure if their habit had worsened, while 5% preferred not to answer this question.
Khader Y. et al./2020 [47] NOS: 5/10	Jordan	368	Dentists (unspecified)	32.9 years ±10.6 66.6% Female March 2020	Attitude in treating potentially infected patients [Non-validated questionnaire]	82.6% of dentists stated that they preferred to avoid treating suspected COVID-19 patients. Facing with a patient sneezing or coughing in their clinics, 43.8% mentioned that they would refer him to the hospital without treating him, 4.6% would refuse to treat the patient, and 49.5% would treat him and ask him to go to the hospital.
Mahdee A.F. et al./2020 [50] NOS: 7/10	Iraq	435	208 General dentists 206 Specialists 21 Consultants	36.51 years ±9.164 49.9% Female From 2 to 23 July 2020	Anxiety [Non-validated questionnaire]	Anxiety of contracting COVID-19 was reported by more than 80% of professionals. The level of anxiety was higher among females and younger dentists, and no significant differences were found in anxiety levels between fields of practice.
Moraes R.R. et al./2021 [56] NOS: 8/10	Brazil	3122	Dentists (unspecified)	38 years ±11 74.5% Female 25.3% Male 0.2% Other From 15 to 24 May 2020	Fear of being infected and pandemic impact on clinical routine [Validated with a pilot study]	90% of dentists were afraid of contracting COVID-19 at work, which was positively related to the number of cases and deaths reported in the state. For each additional 1000 cases/100 deaths, the probability of not working or treating only emergencies increased by 36% and 58%, respectively. The reduction in patients was significantly higher in public clinics.
Nallamothu R. et al./2021 [60] NOS: 7/10	Saudi Arabia, United Arab Emirates, Oman, Kuwait & Qatar	315	Orthodontists	31–40 years (29.84%) 34.9% Female From April to December 2020	Anxiety and depression [Validated with a pilot study]	73.01% of orthodontists reported symptoms of anxiety and depression, and 88.57% informed a negative impact on their income and psychosocial well-being. However, 66.34% stated that, due to increasing of free time, their social life with family and friends had improved.
Owen C. et al./2021 [62] NOS: 4/10	United Kingdom (Wales)	132	Dentists (unspecified)	Age not reported 58.3% Female 40.2% Male 1.5% Other From January to February 2021	(1) Stress [Professional validation] (2) Sleep quality [Non-validated questionnaire]	82% of dentists reported a noticeable increase in stress, and 75% stated they went to work despite not feeling mentally well enough. 40% were drinking alcohol more frequently to deal with stress, and only 11% rested for 6 to 8 h. 91% had already been vaccinated.
Pai S. et al./2021 [64] NOS: 8/10	India	180	65 General dentists 115 Specialists	25–30 years (50.3%) 70.6% Female From April to June 2020	Anxiety and sleep quality [Validated with a pilot study]	25.6% of professionals were anxious during the lockdown, and 57.2% had altered sleep. 36.1% stated that their mental health was more affected than their physical condition, and 28.3% did not noticed changes in both.
Popatrao Patil A. et al./2021 [65] NOS: 4/10	India	300	Dentists (unspecified)	25–45 years Gender not reported From April to June 2021	Stress [Non-validated questionnaire]	81% of dentists felt stressed. Along with the risk of becoming infected and being a source of transmission of COVID-19, professionals had a huge financial burden due to rents, loan pending bills, the cost of preventive kits, and reduced patient flow. 57.3% were already vaccinated.
Plessas A. et al./2021 [67] NOS: 4/10	United Kingdom (England)	38	29 Dentists 9 Dental nurses	Dentists: 17.16 years ±10.89 Dental nurses: 23.12 years ±11.45 68.4% Female From June to August 2020	Frontline experiences [Interview]	The main negative experiences reported in the interviews were feeling frustrated due to fragmented guidance and communication, patient demand outstripping center’s capacity, uncertainty over safety and suffocating personal protective equipment, or lack of commitment to remote video consultations, among others.
Prajapati A.S. et al./2021 [68] NOS: 5/10	India	194	104 General dentists 90 Specialists	Age not reported 43.8% Female From 25 November to 18 December 2020	Anxiety and depression [Professional validation]	49.5% of professionals stated that the personal protective equipment was uncomfortable while performing dental procedures, and only 59.3% used it fully and with an appropriate mask. 38.1% showed symptoms of anxiety and depression, with no significant association with gender or health sector.
Prasetyo Y.T. et al./2021 [69] NOS: 6/10	Indonesia	310	Dentists (unspecified)	27.57 years ±4.54 83.5% Female From 10 August to 1 October 2020	Stress, fear and job satisfaction [Professional validation]	Perceived severity of COVID-19 had significant effects on job stress and the use of personal protective equipment. Moreover, cooperation between management and staff showed a significant association with job stress reduction, which led to greater satisfaction among workers.
Ramesh M. et al./2020 [70] NOS: 7/10	India, Saudi Arabia, UAE, Malaysia, US, United Kingdom & Australia	504	216 General dentists 86 Pediatric dentists 46 Oral pathologists 40 Conservative dentistry 29 Oral surgeons 27 Periodontists 24 Orthodontists 22 Prosthodontists 12 Oral medicine and radiology 2 Community dentistry	25–35 years (55.55%) 49.6% Female From 29 March to 3 April 2020	Stress and fear [Professional validation]	44.4% of dental professionals showed stress about infecting their relatives, and around 28.5% were extremely worried about getting infected with COVID-19 at the workplace, as well as about the information received from the media about the spread of the pandemic.
Sandhu B.K. et al./2021 [73] NOS: 4/10	United Kingdom	40	15 Restorative dentistry 12 Oral surgeons 4 Special care dentistry 9 Other	Age not reported 85% Female July 2020	Impact on the well-being [Focus group discussions]	Main topics highlighted during the interviews included anxiety, safety concerns, family, teamwork and job redeployment. Anxiety and safety were further investigated, identifying discussion of feeling isolated, confusion, or particular concerns about personal protective equipment.
Sarialioglu Gungor A. et al./2021 [75] NOS: 7/10	Turkey	1095	651 General dentists 80 Restorative dentistry 68 Endodontists 67 Prosthodontists 59 Pediatric dentists 57 Periodontists 56 Oral surgeons 51 Orthodontists 6 Radiologists	20–40 years (59.7%) 64.1% Female From 5 to 12 May 2020	Stress and attitude in the treatment of patients [Professional validation]	Stress levels were higher in females, and lower in those dental professionals with more than 20 years of experience. As a preventive measure when returning to work, 86.6% increased the daily patient care intervals, but only 38.4% were using an N95 mask.
Schlenz M.A. et al./2021 [76] NOS: 6/10	Germany	58 Professionals 51 Patients	35 Dentists 23 Dental assistants 51 Dental patients	Professionals: 25–54 years 70.7% Female 19% Male 10.3% Other Patients: 15–54 years 47.1% Female 39.2% Male 13.7% Other From 14 December 2020 to 23 January 2021	Anxiety about becoming infected or infecting others [Professional validation]	Dental assistants reported significantly higher anxiety about COVID-19 (78.9% vs. 27.3% of dentists), and would have preferred only emergency treatment. Patients did not notice any changes in the care received, and perceived high compliance with prevention measures.
Schmidt J. et al./2021 [77] NOS: 5/10	Czech Republic	3674	Dentists (unspecified)	Not reported From 24 February to 9 March 2021	Impact of the pandemic on dental practices (two stages) and patients’ attitudes [Professional validation]	The major reasons for closing dental clinics were a shortage of personal protective equipment (50.5%), an outbreak in the workplace (24.5%), fear of self-infection (24%), and quarantine (20.5%). 47.3% of dentists observed a decreased interest in preventive dental care, and 16.9% noticed worse oral care of patients.
Shetty A. et al./2020 [80] NOS: 7/10	India	405	241 General dentists 69 Endodontists 29 Prosthodontists 13 Orthodontists 13 Periodontists 12 Oral surgeons 11 Pediatric dentists 8 Oral medicine and radiology 5 Oral pathologists 4 Public health dentistry	<35 years (70.4%) 59% Female From March to May 2020	Stress, anxiety and concerns about the COVID-19 infection [Professional validation]	The majority of dental professionals were anxious by the thought of being in a high-risk profession, transmitting the infection to others, and returning to practice after the outbreak. 76.1% stated treating all patients as potentially infected by COVID-19.
Singh Y.P./2021 [81] NOS: 5/10	Saudi Arabia	400	228 Specialists 96 Consultants	20–40 years (57%) 39% Female From March to September 2020	Fear and anxiety [Non-validated questionnaire]	Most professionals were afraid of contracting COVID-19 and transmitting it to their relatives, as well as of getting quarantined. There was a decrease in the volume of patients, and, in general, dentists agreed that it would take more than a year to return to normal, but the majority would not change their profession.
Suryakumari V.B.P. et al./2020 [82] NOS: 6/10	India	307	Dentists (unspecified)	20–40 years (60.26%) 52.77% Female From 9 to 11 May 2020	Fear and anxiety [Non-validated questionnaire]	The mean fear and anxiety score obtained was high (6.57 ± 2.07, in a range from 0 to 9), but 58.31% of dentists showed a low level. Those between 41–60 years of age or with individual practices presented greater scores of fear.
Tokuc B.; Coskunses F.M./2020 [84] NOS: 5/10	Turkey	590	360 General dentists 59 Oral and maxillofacial surgeons 46 Pediatric dentists 30 Prosthodontists 25 Orthodontists 23 Endodontists 22 Periodontists 22 Restorative dentistry 3 Oral and maxillofacial radiologists	20–30 years (48.1%) 55.1% Female From March to April 2020	Anxiety [Non-validated questionnaire]	The mean level of anxiety was 3.35 ± 1.18 (in a range from 0 to 5). 83.1% thought that neither the protective equipment nor the precautions taken would protect them from becoming infected, while only 16% of dental professionals considered them to be really effective.
Turska-Szybka A. et al./2021 [85] NOS: 5/10	Poland	730	Dentists (unspecified)	43.62 years ±11.57 87.8% Female From May to June 2020	Concerns of the risk of infection and anxiety [Validated with a pilot study]	56% of dentists were concerned about the pandemic situation, and 23.6% reported feeling anxious. 42.1% of professionals considered the risk of SARS-CoV-2 infection in the workplace to be very high, and 44.5% planned to become vaccinated as soon as possible.
Tysiąc-Miśta M.; Dziedzic A./2020 [86] NOS: 5/10	Poland	875	Dentists (unspecified)	39.1 years ±11 82.5% Female From 6 to 16 April 2020	Anxiety [Non-validated questionnaire]	71.2% of dentists suspended their clinical practice, with anxiety and uncertainty regarding the generated situation among the decisive factors for this fact. A significant decrease was observed in the number of patients seen per week compared to the period prior to the declaration of the pandemic state.
Uhlen M.M. et al./2021 [87] NOS: 5/10	Norway	1237	590 Dentists 412 Dental assistants 235 Dental hygienists	Age not reported 89.4% Female From May to June 2020	Perception of risk and fear [Professional validation]	58.8% of professionals were working clinically with patients, and most were worried about getting infected (71.9%) or infecting others (85.4%). Some were worried about death (11.7%), perceived that life was threatening (9.8%), or felt loss of control of their lives (8.9 %).
Upadhyay N. et al./2021 [88] NOS: 8/10	India	396	221 General dentists 138 Specialists 37 Consultants	20–30 years (64.4%) 62.1% Female 2020	Fear and anxiety [Professional validation]	55.8% of general dentists reported fear and anxiety. 79.8% were afraid of catching COVID-19 from a patient or co-worker, and 78% were nervous when treating an infected patient.
Wajeeh S. et al./2021 [90] NOS: 8/10	Pakistan	711 Dentists 711 Patients	332 General dentists 286 Specialists 93 Consultants 711 Dental patients	Dentists: 25–45 years (72.5%) 51.2% Female Patients: 25–45 years (46.5%) 46.6% Female From April to July 2021	Dentists: Psychological impact and patient management [Validated with a pilot study] Patients: Satisfaction towards security protocols [Validated with a pilot study]	77.4% of professionals were psychologically affected, with 70.7% showing post-traumatic stress. Many chose to minimize aerosol generation, and using teledentistry in non-emergency cases. 67.9% of patients felt comfortable in the dental office, with 74.5% being satisfied with the clinical services.

All of the studies had a cross-sectional design, except those marked with *.

**Table 3:** Characteristics of studies on dental patients using validated scales.

First Author/Publication Year Quality Assessment	Study Location	Sample Size	Dental Treatment	Characteristics and Period of Data Collection	Variable [Source of Information]	Main Results
Arqub S.A. et al./2021 [96] NOS: 7/10	United States	154	Orthodontic	29.30 years ±12.01 61.7% Female From July to October 2020	(1) Psychological aspects relevant to clinical orthodontics [Validated with a pilot study] (2) Psychological distress [Kessler’s K10]	Average anxiety level was low, and there was a modest association between psychological distress and reduced confidence to resume treatment. 80.51% of patients were extremely pleased with the restrictive protocols, and were very confident in the resumption of treatment.
Arslan I.; Aydinoğlu S./2021 [97] NOS: 8/10	Turkey	250 Children 250 Parents	General (children)	6–12 years (100%) Children 56.8% Girls 31–40 years (56.8%) Parents 87.6% Female 2021	Dental anxiety in children/parents [MCDASf/MDAS]	Anxious children showed a preference for professionals wearing cartoon-printed attire, and for the use of white coats those with low anxiety (*p* = 0.001). Children and parents preferred to receive treatment from a dentist of the same sex as them (*p* < 0.05).
Azevedo Machado B. et al./2022 [99] NOS: 8/10	Brazil	1001	General (children with autism spectrum disorder)	3–18 years (100%) Gender not reported From 1 to 16 September 2020	(1) Fear of COVID-19 [FCV-19S] (2) Attitude towards dental treatment [Validated with a pilot study]	50.35% of parents were very afraid, and 61.64% considered that the pandemic had had a high impact on the daily routine of their children with autism. In addition, 59.34% believed that the use of personal protective equipment could scare these children even more.
Berberoğlu B. et al./2021 [100] NOS: 5/10	Turkey	1439	General	34.8 years ±14.2 58.7% Female From June to September 2020	(1) Previous diagnosis of anxiety/panic attacks or depression [Non-validated questionnaire] (2) Dental anxiety [MDAS] (3) Fear of COVID-19 [COVID-19 Fear and Perception of Control Scale]	The prevalence of dental anxiety was 5.1%, and higher scores were significantly associated with female gender, patients who reported severe pain, and those who felt very or extremely anxious about visiting a dental clinic during the pandemic. Those with previous diagnosis of anxiety/depression (18%) showed an increase in their symptoms.
Carrillo-Diaz M. et al./2021 [104] NOS: 7/10	Spain	124	Orthodontic	41.2 years ±11.4 48.4% Female From 18 March to 15 May 2020	(1) Anxiety [STAI] (2) Fear of COVID-19 [FCV-19S] (3) Dental anxiety [S-DAI]	There was an association between trait anxiety and dental fear with the frequency of spontaneous hand-to-face self-contact of patients in the waiting room. Facial self-contact was higher in women, but it also rose in men as dental fear increased.
Cotrin P. et al./2020 [105] NOS: 6/10	Brazil	354	Orthodontic	35.49 years ±13.93 65.3% Female 2020	Anxiety and impact of quarantine on orthodontic treatment [NRS]	46.3% of patients reported being anxious, with a higher level among females. A significant association was observed between the level of anxiety and the willingness to attend a dental appointment, and the greatest concern of patients was a delay in completion of treatment.
Di Giacomo P. et al./2021 [106] NOS: 5/10	Italy	214	Temporomandibular Disorders	35–55 years (36.7%) 82.7% Female May 2020	(1) Perceived stress [PSS] (2) Psycho-physical impact [Non-validated questionnaire]	The most prevalent category was ‘moderate stress’, and patients attributed a medium/low impact to the pandemic. The intensity of orofacial symptoms during this period was even lower than before. The age group was statistically significant regarding the COVID-19 pandemic impact score.
Folayan M.O. et al./2021 [108] NOS: 9/10	Nigeria	966	General	31.25 years ±9.90 49.6% Female From 21 June to 6 August 2020	(1) Psychological well-being [WHO-5 Well-Being Index] (2) Anxiety and depression [Hospital Anxiety and Depression Scale] (3) Perceived social support [Multidimensional Scale of Perceived Social Support]	Generalized anxiety significantly accounted for approximately 12% of the total effect of wellbeing on decreased tooth brushing, and for 70% of the total effect of wellbeing on the presence of oral ulcers.
González-Olmo M.J. et al./2021 * [110] NOS: 8/10	Spain	961	General	38.4 years ±16.1 58.2% Female Time 0: from 1 to 8 March 2020 Time 1: from 4 to 11 May 2020	(1) Perceived vulnerability to disease [PVDS] (2) Fear of COVID-19 [FCV-19S] (3) Avoidance behavior towards dental clinics [Non-validated questionnaire]	Infectability and germ aversion scores were higher after lockdown completion (*p* < 0.01). 24.5% of respondents stated that they would not go to the dentist for fear of COVID-19, and those older than 60 years were 8 times more likely not to attend.
González-Olmo M.J. et al./2020 [111] NOS: 5/10	Spain	1008	General	18–83 years (100%) 58.5% Female From 1 to 8 March 2020	(1) Perceived vulnerability to disease [PVDS] (2) Fear of going to the dentist and perception of risk of contagion (certain places/dental clinics) [Non-validated questionnaire]	Significant differences by sex were found on the germ aversion subscale and in the risk of infection in the waiting room, tooth extraction, endodontics, and fillings, with women considering the risk to be higher.
Ibrahim M.S. et al./2021 [113] NOS: 9/10	Saudi Arabia	826	General	38.84 years ±13.29 65.5% Female From May to June 2020	(1) Fear of COVID-19 [FIVE] (2) Fear to seek dental care [Validated with a pilot study] (3) Perceived risk of infection [Validated with a pilot study]	Fear of seeking dental care was significantly higher among females, the 35–44 age group, those who perceived moderate and high risk of COVID-19 infection in dental clinics, and individuals with untreated dental conditions.
Luo Y./2021 [114] NOS: 7/10	United States	3246	General	>65 years (58.2%) 56.8% Female From June 2020 to February 2021	(1) Depression [CIDI-SF] (2) Delayed care and depression [Non-validated questionnaire] (3) Pandemic stressors [Non-validated questionnaire]	8.8% of participants had depression during the pandemic. Delayed dental care and self-reported pain were positively associated with depression among both middle-aged adults and older than 65 years, whereas financial difficulties were associated with depression in middle-aged adults.
Martina S. et al./2021 [116] NOS: 7/10	Italy	1566	Orthodontic	18–39 years (61.1%) 54.8% Female May 2020	(1) Anxiety over going to the dentist and perception of risk [Non-validated questionnaire] (2) Anxiety and depression [PHQ-4]	55.3% of patients considered that the risk of infection was higher in dental practice, which was associated with female gender, individuals over 60 years of age and high levels of distress. 57.1% felt comfortable going back to the dentist, and 84% were willing to continue their treatment.
Moghadam M.G. et al./2021 [118] NOS: 7/10	Iran	324	Orthodontic	32.43 years 76.9% Female From 21 to 27 June 2020	(1) Perceived anxiety [NRS] (2) Attendance to orthodontic appointments [Non-validated questionnaire]	72% of patients reported feeling calm about the pandemic and its consequences. 74% stated that they would attend their orthodontic appointment in case of emergency, and 41% said that their greatest concern was a possible delay in completing treatment.
Nazir M. et al./2021 [120] NOS: 9/10	Saudi Arabia	606	General	30.49 years ±12.01 40.6% Female From June to July 2020	Dental fear [DFS]	22.6% of those surveyed showed a high fear level. About 36.8% stated that they preferred to visit a dental clinic only in case of emergency, and 46.2% reported visiting the dentist in less than 6 months. Female gender, the time of last visit, and dental pain were significant predictors of dental fear.
Olivieri J.G. et al./2021 * [121] NOS: 8/10	Spain	96	Root Canal	47.3 years ±16.3 55.2% Female Strict confinement: from 14 March to 21 May 2020 Partial confinement: from 25 May to 18 June 2020	(1) Dental anxiety [MDAS] (2) Fear of endodontic treatment [Non-validated questionnaire]	Anxiety scores decreased when restrictive measures were relaxed; nevertheless, fear scores increased. A previous bad dental experience resulted in higher levels of anxiety and fear, and an increase in heart rate was observed in patients with higher scores for both variables.
Pylińska-Dąbrowska D. et al./2020 * [127] NOS: 5/10	Poland	175	Oral Surgery	18–35 years (50.9%) 61.7% Female From November 2019 to September 2020	(1) Fear of COVID-19 [Non-validated questionnaire] (2) Dental anxiety [MDAS] (3) Quality of life [ED-5Q/EQ-VAS]	21.9% of patients who underwent oral surgery procedures had higher anxiety when compared to the pre-pandemic group. There was an increase in moderate dental anxiety and a decrease of 10 percentage points in the quality of patients’ health.
Quan S. et al./2021 [128] NOS: 7/10	China	1078	Orthodontic	22.59 years ±8.277 72.9% Female From 20 February to 5 March 2020	(1) Anxiety [SAS] (2) Attitude towards dental treatment [Non-validated questionnaire]	Female and elder patients, and those who experienced orthodontic emergencies during the pandemic had higher anxiety levels. 33.67% reported orthodontic problems, mostly related to treatments with fixed or removable appliances.
Samuel S.R. et al./2021 [129] NOS: 9/10	India	2462	General	42.7 years 46% Female From March to June 2020	(1) Fear of COVID-19 [FCV-19S] (2) Psychological distress [Professional validation] (3) Oral health impact [OHIP-14] (4) Access to dental care/medicines [Professional validation]	Greater self-reported pain, suffering from pain for more than 15 days, and higher fear and psychological distress scores were associated with poorer quality of life regarding oral health. In addition, 95% of participants reported closure of nearby clinics and 73.4% informed lack of analgesics.
Sari A.; Bilmez Z.Y./2021 [130] NOS: 9/10	Turkey	1227	General	18–25 years (27.7%) 56.6% Female From 1 August to 1 October 2020	(1) Fear of COVID-19 [FCV-19S] (2) Tendency to visit the dentist [Non-validated questionnaire]	Respondents with higher fear scores began brushing and using oral care products more regularly, and reported increased consumption of sugary food. Despite the high prevalence of dental problems, these patients hesitated to visit the dentist.
Wen Y.F. et al./2021 [134] NOS: 6/10	China	636	General	30–59 years (55.4%) 55.7% Female From February to March 2020	(1) Attitude towards dental treatment [Professional validation] (2) Anxiety [IAS]	Inability to wear masks during treatment was the most closely associated factor with the general pattern of participants dental attendance. Unnecessary dental avoidance was associated with perceived risk of infection and personal traits such as trust and anxiety.
Wu Y. et al./2021 [135] NOS: 7/10	China	1241	Temporomandibular Disorders	26.41 years ±8.225 71.2% Female From 19 to 29 February 2020	Psychological distress [Kessler’s K10]	These patients reported higher levels of anxiety and depression than orthodontic patients and the general population. Female gender, younger age, having close contact with people from Hubei, or a higher self-rated infection possibility were negatively affecting their psychological status.
Xiong X. et al./2020 [136] NOS: 6/10	China	458	Orthodontic	24.78 years ±6.33 77.3% Female From 20 to 22 February 2020	(1) Psychological distress [Kessler’s K10] (2) Orthodontic related mental state [Professional validation]	The prevalence of psychological distress was 38%, and higher odds ratios were associated with female gender, missed appointments, and residing in Hubei. The type of orthodontic appliance was positively associated with anxiety, due to its implication in a longer treatment duration.
Yavan M.A./2021 [137] NOS: 6/10	Turkey	241	Orthodontic	17.73 years ±3.27 73.4% Female June 2020	(1) Anxiety [STAI] (2) Treatment related concerns [Non-validated questionnaire]	Significantly higher levels of anxiety were observed in women, and there was a positive correlation with age. The most anxious patients considered dental clinics as risky environments for the spread of SARS-CoV-2, and preferred to resume their treatment once the pandemic was over.

ASDS: Acute Stress Disorder Scale; GAD-7: Generalized Anxiety Disorder-7; CPDI: COVID-19 Peritraumatic Distress Index; DASS-21: Depression, Anxiety and Stress Scales-21; FCV-19S: Fear of COVID-19 Scale; GHQ-28: General Health Questionnaire-28; GP-CORE: General Population-Clinical Outcomes in Routine Evaluation; IES-R: Impact of Event Scale-Revised; MBI: Maslach Burnout Inventory; MSPSS: Multidimensional Scale of Perceived Social Support; NSESSS: National Stressful Events Survey PTSD Short Scale; NRS: Numeric Rating Scale; PHQ-2/-4/-9: Patient Health Questionnaire-2/-4; PSQI: Pittsburgh Sleep Quality Index; PSS: Perceived Stress Scale; PSS-10/-14: Perceived Stress Scale-10/-14 PSS-SR: Post-traumatic stress disorder Symptom Scale-Self Report; PTGI-SF: Post-Traumatic Growth Inventory-Short Form; SMDA: Severity Measure for Depression-Adult; STAI: State-Trait Anxiety Inventory. All of the studies had a cross-sectional design, except those marked with *.

**Table 4:** Characteristics of studies on dental patients using non-validated scales.

First Author/Publication Year Quality Assessment	Study Location	Sample Size	Dental Treatment	Characteristics and Period of Data Collection	Variable [Source of Information]	Main Results
Abdulkareem A.A. et al./2021 [95] NOS: 7/10	Iraq, Egypt & Jordan	3782	General	27.99 years ±9.44 80.5% Female From 10 to 24 August 2020	(1) Attitude towards dental treatment [Validated with a pilot study] (2) Fear of COVID-19 [Validated with a pilot study]	87% of those surveyed chose to leave the dental clinics due to fear of contracting the SARS-CoV-2, with 79% considering the clinical environment as ‘high-risk’. Female and subjects over 25 years of age reported significantly higher levels of fear.
Ashraf A. et al./2021 [98] NOS: 6/10	India	216	General	29.09 years ±8.83 64.8% Female From 15 to 31 May 2020	Anxiety [Non-validated questionnaire]	More than 70% of respondents were concerned about the pandemic, and 46.3% felt stressed for themselves and for their close ones. The prevalence of psychological distress was 42.6%, and 42.1% reported having inappropriate social behavior upon the fear of contracting the SARS-CoV-2.
Blumer S. et al./2021 [101] NOS: 6/10	Israel	361	General (children)	42.0 years ±6.2 87% Female From March to April 2020	(1) Parental functioning and mental resilience [Validated with a pilot study] (2) Children’s mental stress [Validated with a pilot study] (3) Access to medical treatments [Validated with a pilot study]	Most parents had adapted well to the changes imposed by lockdown, reporting that they and their children had low levels of anxiety and high mental resilience. 60% had difficulties in accessing dental care, and 60.9% reported an increase in the consumption of snacks and sweets.
Bustati N.; Rajeh N./2020 [102] NOS: 6/10	Syria	388	Orthodontic	20.4 years ±4.0 75% Female 2020	Missing dental appointments [Professional validation]	69% of patients stated that the closure of the clinic was the main reason for missing their appointments, and 16% did not attend due to fear of COVID-19. 84% had fixed appliances and would have called their orthodontist in case of problems, just like the group of transparent aligners.
Campagnaro R. et al./2020 [103] NOS: 7/10	Brazil	1003 Parents	General (children)	36.6 years ±6.97 (97.2%) Gender not reported From 12 May to 9 June 2020	Fear of getting infected and attitude towards dental treatment [Non-validated questionnaire]	A significant association was observed between parents’ willingness to take their children to the dentist with the level of fear, and 66.6% of parents would only seek urgent dental care. 61.5% stated that their diet had changed during the pandemic period.
Farsi D.; Farsi N./2021 [107] NOS: 6/10	Saudi Arabia	833	General (children)	31–40 years (53.1%) 100% Female June 2020	Willingness and barriers to visiting a dentist [Professional validation]	83% of mothers did not request dental care for their children, and 24% of those who already had an appointment did not allow their children to attend. 80% reported that the main barrier to visit the dental clinic was fear of being infected by SARS-CoV-2 from someone there.
Gallegati S. et al./2021 [109] NOS: 6/10	Italy	1003	General	25–34 years (32.4%) 60.7% Female From 11 to 18 May 2020	Willingness to visit a dentist based on preferred COVID-19 information source [Professional validation]	The more informed the participants were, the higher was the risk of missing dental appointments. The two most reliable communication channels were journals and websites for healthcare professionals. Women were more active in collecting information and relying on less secure channels.
Hajek A. et al./2021 [112] NOS: 7/10	Germany	974	General	45.9 years ±16.5 51.1% Female From 21 to 22 July 2020	Postponing dental visits [Validated with a pilot study]	22% of those surveyed stated that they had postponed dental appointments: 72% postponed a check-up, 19.6% a planned therapy, and 8.4% did so despite the pain. The probability of postponement was positively associated with being younger and a higher affectation by COVID-19.
Majeed M.M. et al./2021 [115] NOS: 7/10	Pakistan	461	General	31–40 years (36.4%) 41% Female May 2020	Fear and anxiety [Non-validated questionnaire]	63.6% of respondents reported fear of visiting the dentist, and 66.2% thought that they would become infected in the dental office. Women were more anxious, as well as participants with an infected family member. Significant differences were found according to age and educational level.
Moffat R.C. et al./2020 [117] NOS: 6/10	United States	452	General	40.7 years ±11.2 43.8% Female 55.3% Male 0.9% Other May 2020	(1) Perceived susceptibility and risk of infection from attending a dental appointment [Non-validated questionnaire] (2) Conditions for a safe return to the dental office [Non-validated questionnaire]	Older age was positively associated with perceived susceptibility to contracting COVID-19 in a dental clinic. Confirmation by public health officials of the safety of dental settings was reported as the most important factor for returning to routine dental visits.
Nardi G.M. et al./2021 [119] NOS: 6/10	Italy	1135	General	20–40 years (51.8%) 68.9% Female From 1 November to 30 December 2020	Attitude towards dental treatment [Non-validated questionnaire]	The greatest concern of participants was infecting a family member. The restrictive measures that forced people to stay at home led to an increased consumption of cariogenic foods. Patients felt safe when visiting the dentist, and asked for introducing telemedicine in similar situations in the future.
Olszewska A.; Rzymski P./2020 [122] NOS: 5/10	Poland	20 (2018) 25 (2020) And their caregivers	General (children)	2018 5.6 years ±1.1 Girls 4.5 years ±0.8 Boys 50% Girls 2020 5.3 years ±0.9 Girls 5.1 years ±1.1 Boys 40% Girls From 24 March to 30 April 2020	Children: Emotional state [Faces Mood Scale] Caregivers: Anxiety [Non-validated questionnaire]	Children who required dental care during quarantine did not show a significantly higher level of anxiety as compared to the pre-pandemic control group. Caregiver anxiety levels were higher in the pandemic group, revealing stronger correlations with dental anxiety in children.
Papautsky E.L. et al./2021 [123] NOS: 6/10	United States	2570	General	37.3 years ±12.6 95.6% Female 3.2% Male 1.2% Other From 5 April to 5 May 2020	Interruptions in dental care [Professional validation]	The main reported barrier was fear of infection (33.6%). 47.7% of respondents stated experiencing health care delays, mainly regarding dental (38.1%), preventive (29.2%) and diagnostic (16.4%) care. Age, female gender, and concern about general health were significantly positively associated with these delays.
Pasiga B.D./2020 [124] NOS: 5/10	Indonesia	285	General	29.91 years 64.9% Female From 21 May to 13 June 2020	Fear of dental care [Professional validation]	The knowledge of participants about the transmission of SARS-CoV-2 in dental practice was 79.9%, and the fear of caring during the pandemic was 31.85%. Statistical analysis showed a significant association between both factors.
Peloso R.M. et al./2020 [125] NOS: 6/10	Brazil	595	263 Orthodontic 65 Oral rehabilitation 26 Restorative or others 241 No treatment	38.21 years ±13.94 69.2% Female April 2020	Concerns about attending dental appointments and impact on orthodontic treatment [Non-validated questionnaire]	Most patients were on active orthodontic treatment, and said that they would attend their appointments; nevertheless, those who were not receiving treatment would not visit or would visit only in an emergency. Men showed calmer, and patients on active treatment were concerned about a possible delay.
Phadraig C.M.G. et al./2021 [126] NOS: 5/10	Worldwide Most from Europe (40.4%)	436	General (people with disabilities)	30–50 years (55.5%) 70.6% Female 29.1% Male 0.2% Other From 10 to 31 July 2020	Changes and restrictions on dental practice [Professional validation]	A significant reduction was observed during and after the lockdown regarding the number of dentists who were treating people with disabilities and the disabled patients seen per week. Lack of pharmacological support increased from 22% pre-lockdown to 61% during lockdown, and a persistent 44% after lockdown.
Tatar N.; Karabas A./2021 [131] NOS: 4/10	Turkey	135	Prosthodontic	18–30 years (40%) 54.8% Female From July to October 2020	Fear of contamination during dental treatment [Non-validated questionnaire]	24% of patients requiring prosthodontic treatments considered it to be risky, especially in the 31–60 age group. Those participants with a history of SARS-CoV-2, and/or with a member of their social circle with a history of the virus or who had died, were unwilling to receive dental care.
Vanka S. et al./2021 [132] NOS: 5/10	Saudi Arabia	283	General	Not reported 2020	Dental services utilization [Validated with a pilot study]	51.6% of respondents were unaware of the precautions to be taken when visiting the dentist. The main barrier to the use of dental services was fear of COVID-19 transmission (58.2%); on the other hand, safety was the first reason for using teledentistry (27.2%).
Vohra P. et al./2021 [133] NOS: 5/10	India	511	General	20–30 years (38.1%) 29.8% Female 66.9% Male 3.3% Other From November 2020 to February 2021	Fear and anxiety visiting the dentist [Professional validation]	62.4% of those surveyed were stressed, and showed anxiety regarding their visit for dental check-up and treatment, as well as fear of contracting the infection due to the dental procedures. Although 26% were not willing for treatment, 62.4% were willing to receive dental treatment post-vaccination.

All of the studies had a cross-sectional design.

## Data Availability

Not applicable.

## Supplementary Materials

The following supporting information can be downloaded at: https://www.mdpi.com/article/10.3390/ijerph192316216/s1, Supplementary Materials S1: Modified Newcastle-Ottawa Scale (NOS). Adaptation for cross-sectional studies based on surveys; Table S1: Number of studies from each country; Table S2: Vaccination and pandemic moment of the studies on dental professionals; Table S3: Vaccination and pandemic moment of the studies on dental patients; Table S4: Quality assessment of studies on dental professionals using the modified Newcastle-Ottawa Scale; Table S5: Quality assessment of studies on dental patients using the modified Newcastle-Ottawa Scale.
