# Supplementary material for: Psychological Impact of COVID-19 in the Setting of Dentistry: A Review Article
Source: Int J Environ Res Public Health. 2022 Dec 4;19(23):16216. doi: 10.3390/ijerph192316216 (PMC9739953; doi:10.3390/ijerph192316216)
Supplement: Supplementary file 1 [file ijerph-19-16216-s001.zip › ijerph-1997680-supplementary.pdf]

Supplementary Material S1. Modified Newcastle-Ottawa Scale (NOS). Adaptation for cross-sectional studies based on surveys

Selection (Maximum 3 stars)

1. **Representativeness of the sample**

   a) Truly representative of the average in the target population. ✳ (all subjects or random sampling)
   b) Somewhat representative of the average in the target population. ✳ (non-random sampling)
   c) Selected group of users.
   d) No description of the sampling strategy.

2. **Sample size**

   a) Justified and satisfactory. ✳
   b) Not justified.

3. **Non-respondents**

   a) Comparability between respondents and non-respondents characteristics is established, and the response rate is satisfactory. ✳
   b) The response rate is unsatisfactory, or the comparability between respondents and non-respondents is unsatisfactory.
   c) No description of the response rate or the characteristics of the responders and the non-responders.

Comparability (Maximum 2 stars)

1. The subjects in different outcome groups are comparable, based on the study design or analysis. Confounding factors are controlled.

   a) The study controls for the most important factor (select one). ✳
   b) The study control for any additional factor. ✳

Outcome (Maximum 5 stars)

1. **Assessment of the outcome I**

   a) Independent blind assessment. ✳✳
   b) Record linkage. ✳✳
   c) Self-report. ✳
   d) No description.

2. **Assessment of the outcome II**

   a) Validated measurement tool. ✳✳
   b) Non-validated measurement tool, but the tool is available or described. ✳
   c) No description of the measurement tool.

4. **Statistical test**

   a) The statistical test used to analyze the data is clearly described and appropriate, and the measurement of the association is presented, including confidence intervals and the probability level (p value). ✳

b) The statistical test is not appropriate, not described or incomplete.

Score

Very Good quality: 9 · 10 stars.

Good quality: 7 · 8 stars.

Satisfactory quality: 5 · 6 stars.

Unsatisfactory quality: <5 stars.

This scale has been adapted from the Newcastle-Ottawa Quality Assessment Scale for cohort studies. An additional adaptation has been done for this study: the point 4 of 'Selection' section is referred to the validity of measurement tool. The studies selected in this review used the tools to evaluate the psychological impact as a result (consequence of the COVID-19 pandemic) and not as an exposure. So, this point was moved to 'Outcome' section (Assessment of the outcome II).

Table S1. Number of studies from each country.

Country	Total number of studies	Studies on dental professionals	Studies on dental patients
Australia	1	1	0
Brazil	10	6	4
Cambodia	1	1	0
Canada	1	1	0
China	8	4	4
Czech Republic	1	1	0
Ecuador	1	1	0
Egypt	2	1	1
France	1	1	0
Germany	5	4	1
Hungary	1	1	0
India	20	17	3
Indonesia	2	1	1
Iran	3	2	1
Iraq	2	1	1
Israel	2	1	1
Italy	11	7	4
Jordan	2	1	1
Kuwait	1	1	0
Malaysia	2	2	0

Table S1. Continued.

Country	Total number of studies	Studies on dental professionals	Studies on dental patients
Nigeria	1	0	1
Norway	1	1	0
Oman	1	1	0
Pakistan	7	6	1
Peru	1	1	0
Poland	4	2	2
Qatar	1	1	0
Romania	1	1	0
Russia	1	1	0
Saudi Arabia	12	8	4
Spain	5	1	4
Syria	1	0	1
Taiwan	1	1	0
Trinidad & Tobago	1	1	0
Turkey	13	8	5
United Arab Emirates	2	2	0
United Kingdom	9	9	0
United States	6	2	4
Venezuela	1	1	0

Table S2. Vaccination and pandemic moment of the studies on dental professionals.

First author / Publication year	Study location	Period of data collection	Vaccination process started (Yes/No)	Pandemic moment
Ahmed, M.A. et al. / 2020 [15]	30 countries worldwide<br>Most from Pakistan (30.8%)	From 10th to 17th March 2020	No	No cases or deaths in Pakistan
Ajwa, N. et al. / 2020 [16]	Saudi Arabia	From June to August 2020	No	Second wave; high mortality
Aldhuwayhi S. et al. / 2021 [17]	Saudi Arabia	2020	No	Unknown
Alencar C. de M. et al. / 2021 [18]	Brazil	From 11th to 30th July 2020	No	First wave; high mortality
Alkhalifah F.N. et al. / 2021 [19]	Saudi Arabia	From 15th to 28th May 2020	No	First wave; rising mortality
Aly, M.M.; Elchaghaby, M.A. / 2020 [20]	Egypt	2020	No	Unknown
Amato A. et al. / 2021 [21]	Italy	From April 26th to May 3rd 2020	No	First wave; high mortality
Aurlene N. et al. / 2021 [22]	India	From June to September 2020	No	First wave; high mortality
Balkaran R. et al. / 2021 [23]	10 Caribbean countries<br>Most from Trinidad and Tobago (77.6%)	From December 2020 to March 2021	Yes	Few cases or deaths in Trinidad and Tobago
Bellini, P. et al. / 2020 [24]	Italy	From 2nd to 29th April 2020	No	First wave; high mortality
Campos J.A.D.B. et al. / 2021 [25]	Brazil	From May 18th to June 13th 2020	No	Few cases or deaths
Çelik O.E.; Cansever İ.H. / 2021 [26]	Turkey	From September 30th to October 20th 2020	No	Second wave; rising mortality
Chakraborty, T. et al. / 2020 [27]	India	From 1st to 10th May 2020	No	Few cases or deaths

Table S2. Continued.

First author / Publication year	Study location	Period of data collection	Vaccination process started (Yes/No)	Pandemic momento
Chaudhary F.A. et al. / 2021 [28]	Pakistan	From April to July 2020	No	First wave; high mortality
Chen Y.; Li W. / 2021 [29]	China	From 3rd to 10th April 2020	No	Few cases or deaths
Cheng H-C. et al. / 2021 [30]	Taiwan	May 2018 / April 2020	No	Few cases or deaths
Collin, V. et al. / 2021 [31]	United Kingdom	From 22nd to 27th May 2020	No	First wave; decreasing mortality
Consolo, U. et al. / 2020 [32]	Italy	From 2nd to 21st April 2020	No	First wave; high mortality
Cotrin, P. et al. / 2020 [33]	Brazil	2020	No	Unknown
De Stefani, A. et al. / 2020 [34]	Italy	From 11th to 18th April 2020	No	First wave; high mortality
Dreher A. et al. / 2021 [35]	Germany	From 7th to 14th April 2020	No	First wave; high mortality
Estrich C.G. et al. / 2021 [36]	United States	From September 29th to October 8th 2020	No	Few cases or deaths
Fairozekhan A.T. et al. / 2021 [37]	7 Asian countries<br>Most from India (47%)	From April 15th to May 5th 2020	No	Few cases or deaths in India
Ortega-López M.F. et al. / 2021 [38]	14 Latin American countries<br>Most from Peru (23.7%), Ecuador (21.6%), Venezuela (12,2%) and Brazil (8,6%)	2020	No	Unknown
Gasparro, R. et al. / 2020 [39]	Italy	From April 17th to May 3rd 2020	No	First wave; high mortality

Table S2. Continued.

First author / Publication year	Study location	Period of data collection	Vaccination process started (Yes/No)	Pandemic moment
Humphris G. et al. / 2021 [40]	United Kingdom (Scotland)	From August to October 2020	No	Few cases or deaths
Iorga M. et al. / 2021 [41]	Romania	From November 18th to December 5th 2020	No	First wave; high mortality
Kale P. et al. / 2021 [42]	India	From September to October 2020	No	First wave; high mortality
Kamal A.T. et al. / 2021 [43]	Pakistan	From July 20th to August 5th 2020	No	Few cases or deaths
Kamran R. et al. / 2021 [44]	Pakistan	From 16th to 20th June 2020	No	First wave; high mortality
Karayürek, F. et al. / 2021 [45]	Turkey	2020	No	Unknown
Karobari M.I. et al. / 2021 [46]	India, Malaysia, Saudi Arabia, Pakistan, United Kingdom & Cambodia	From February to May 2021	Yes	India: second wave; high mortality<br>Malaysia: first wave; few cases or deaths<br>Saudi Arabia: second wave; low mortality<br>Pakistan: few cases or deaths<br>UK: high mortality<br>Cambodia: first wave; high mortality
Khader, Y. et al. / 2020 [47]	Jordan	March 2020	No	Few cases or deaths
Kirli M.C.; Kirli U. / 2021 [48]	Turkey	2020: Before and after restarting high-risk procedures	No	Before and after restarting high-risk procedures
Labban N. et al. / 2021 [49]	Saudi Arabia	From July to August 2020	No	Second wave; decreasing mortality
Mahdee, A.F. et al. / 2020 [50]	Iraq	From 2nd to 23rd July 2020	No	Few cases or deaths
Martina, S. et al. / 2020 [51]	Italy	From 1st to 6th May 2020	No	First wave; high mortality

Table S2. Continued.

First author / Publication year	Study location	Period of data collection	Vaccination process started (Yes/No)	Pandemic moment
Mekhemar M. et al. / 2021 [52]	Germany	From July 2020 to January 2021	Yes	Second wave; high mortality
Mekhemar M. et al. / 2021 [53]	Germany	From July to November 2020	No	Second wave; high mortality
Mijiritsky, E. et al. / 2020 [54]	China, India, Israel, Italy & United Kingdom	From March 30th to April 12th 2020	No	First wave and high mortality in Italy & UK
Mishra, S. et al. / 2020 [55]	India	Phase I: from 20th to 25th March 2020<br>Phase II: from 25th to 30th April 2020	No	Phase I & II: few cases or deaths
Moraes R.R. et al. / 2021 [56]	Brazil	From 15th to 24th May 2020	No	Few cases or deaths
Mulla, S. et al. / 2020 [57]	India	From March 25th to May 25th 2020	No	First wave; low mortality
Nagarajappa R. et al. / 2021 [58]	India	From June to August 2020	No	First wave; high mortality
Nair, A.K.R. et al. / 2020 [59]	India	From 8th to 16th April 2020	No	Few cases or deaths
Nallamothu R. et al. / 2021 [60]	Saudi Arabia, United Arab Emirates, Oman, Kuwait & Qatar	From April to December 2020	No	8 months in multiple countries
Olivieri, J.G. et al. / 2021 [61]	Spain	Phase I: from March 27th to May 21st 2020<br>Phase II: from May 26th to June 18th 2020	No	Phase I: first wave; high mortality<br>Phase II: first wave; low mortality
Owen C. et al. / 2021 [62]	United Kingdom (Wales)	From January to February 2021	Yes	Second wave; peak mortality
Özarslan M.; Caliskan S. / 2021 [63]	Turkey	From March 9th to May 20th 2020	No	First wave; low mortality
Pai S. et al. / 2021 [64]	India	From April to June 2020	No	First wave; low mortality

Table S2. Continued.

First author / Publication year	Study location	Period of data collection	Vaccination process started (Yes/No)	Pandemic moment
Popatrao Patil A. et al. / 2021 [65]	India	From April to June 2021	Yes	Second wave; high mortality
Peixoto K.O. et al. / 2021 [66]	Brazil	From May to June 2020	No	First wave; rising mortality
Plessas A. et al. / 2021 [67]	United Kingdom (England)	From June to August 2020	No	Few cases or deaths
Prajapati A.S. et al. / 2021 [68]	India	From November 25th to December 18th 2020	No	First wave; low mortality
Prasetyo Y.T. et al. / 2021 [69]	Indonesia	From August 10th to October 1st 2020	No	Few cases or deaths
Ramesh, M. et al. / 2020 [70]	India, Saudi Arabia, UAE, Malaysia, US, United Kingdom & Australia	From March 29th to April 3rd 2020	No	First wave and high mortality only in UK
Ranka M.S.; Ranka S.R. / 2021 [71]	United Kingdom	June 2020	No	First wave; low mortality
Salehiniya H.; Abbaszadeh H. / 2021 [72]	Iran	From 2nd to 14th May 2020	No	Few cases or deaths
Sandhu B.K. et al. / 2021 [73]	United Kingdom	July 2020	No	Few cases or deaths
Sarapultseva, M. et al. / 2021 [74]	Russia	From 1st to 20th September 2020	No	Few cases or deaths
Sarialioglu Gungor A. et al. / 2021 [75]	Turkey	From 5th to 12th May 2020	No	First wave; decreasing mortality
Schlenz M.A. et al. / 2021 [76]	Germany	From December 14th 2020 to January 23rd 2021	Yes	Second wave; high mortality
Schmidt J. et al. / 2021 [77]	Czech Republic	From February 24th to March 9th 2021	Yes	Questions about first wave (few cases or deaths /high mortality)

Table S2. Continued.

First author / Publication year	Study location	Period of data collection	Vaccination process started (Yes/No)	Pandemic moment
Serota K.S. et al. / 2021 [78]	Hungary	2021	Yes	Unknown
Shacham, M. et al. / 2021 [79]	Israel	From March 30th to April 10th 2020	No	Few cases or deaths
Shetty, A. et al. / 2020 [80]	India	From March to May 2020	No	First wave; low mortality
Singh Y.P. / 2021 [81]	Saudi Arabia	From March to September 2020	No	First wave; low mortality
Suryakumari, V.B.P. et al. / 2020 [82]	India	From 9th to 11th May 2020	No	First wave; low mortality
Tao J. et al. / 2021 [83]	China	From 3rd to 10th April 2020	No	Few cases or deaths
Tokuc, B.; Coskunses, F.M. / 2020 [84]	Turkey	From March to April 2020	No	First wave; rising mortality
Turska-Szybka A. et al. / 2021 [85]	Poland	From May to June 2020	No	Few cases or deaths
Tysiąc-Miśta, M.; Dziedzic, A. / 2020 [86]	Poland	From 6th to 16th April 2020	No	Few cases or deaths
Uhlen M.M. et al. / 2021 [87]	Norway	From May to June 2020	No	Few cases or deaths
Upadhyay N. et al. / 2021 [88]	India	2020	No	Unknown
Uziel N. et al. / 2021 [89]	Israel, Canada & France	From April 18th to June 13th 2020	No	Few cases or deaths in Israel & Canada<br>First wave and high mortality in France
Wajeeh S. et al. / 2021 [90]	Pakistan	From April to July 2021	Yes	Third and fourth wave

Table S2. Continued.

First author / Publication year	Study location	Period of data collection	Vaccination process started (Yes/No)	Pandemic moment
Yilmaz, H.N.; Ozbilen, E.O. / 2020 [91]	Turkey	From 6th to 15th June 2020	No	Few cases or deaths
Yılmaz M. et al. / 2021 [92]	Turkey	From April 1st to May 1st 2020	No	First wave; peak mortality
Zeidi I.M.; Zeidi B.M. / 2021 [93]	Iran	From April 4th to July 18th 2020	No	Few cases or deaths
Zhao, S. et al. / 2020 [94]	China	From 2nd to 13th May 2020	No	Few cases or deaths

Table S3. Vaccination and pandemic moment of the studies on dental patients.

First author / Publication year	Study location	Period of data collection	Vaccination process started (Yes/No)	Pandemic moment
Abdulkareem A.A. et al. / 2021 [95]	Iraq, Egypt & Jordan	From 10th to 24th August 2020	No	Rising mortality only in Iraq
Arqub S.A. et al. / 2021 [96]	United States	From July to October 2020	No	Second wave; low mortality
Arslan I.; Aydinoğlu S. / 2021 [97]	Turkey	2021	Unknown	Unknown
Ashraf A. et al. / 2021 [98]	India	From 15th to 31st May 2020	No	First wave; low mortality
Azevedo Machado B. et al. / 2022 [99]	Brazil	From 1st to 16th September 2020	No	First wave; decreasing mortality
Berberoğlu B. et al. / 2021 [100]	Turkey	From June to September 2020	No	Few cases or deaths
Blumer S. et al. / 2021 [101]	Israel	From March to April 2020	No	Few cases or deaths
Bustati, N.; Rajeh, N. / 2020 [102]	Syria	2020	No	Unknown
Campagnaro, R. et al. / 2020 [103]	Brazil	From May 12th to June 9th 2020	No	First wave; rising mortality
Carrillo-Diaz M. et al. / 2021 [104]	Spain	From March 18th to May 15th 2020	No	First wave; high mortality
Cotrin, P. et al. / 2020 [105]	Brazil	2020	No	Unknown
Di Giacomo P. et al. / 2021 [106]	Italy	May 2020	No	First wave; high mortality
Farsi D.; Farsi N. / 2021 [107]	Saudi Arabia	June 2020	No	Second wave; peak mortality

Table S3. Continued.

First author / Publication year	Study location	Period of data collection	Vaccination process started (Yes/No)	Pandemic moment
Folayan M.O. et al. / 2021 [108]	Nigeria	From June 21st to August 6th 2020	No	First wave; high mortality
Gallegati S. et al. / 2021 [109]	Italy	From 11th to 18th May 2020	No	First wave; high mortality
González-Olmo M.J. et al. / 2021 [110]	Spain	Time 0: from 1st to 8th March 2020<br>Time 1: from 4th to 11th May 2020	No	Time 0: no deaths<br>Time 1: first wave; decreasing mortality
González-Olmo, M.J. et al. / 2020 [111]	Spain	From 1st to 8th March 2020	No	First wave; no deaths
Hajek, A. et al. / 2021 [112]	Germany	From 21st to 22nd July 2020	No	Few cases or deaths
Ibrahim M.S. et al. / 2021 [113]	Saudi Arabia	From May to June 2020	No	First wave; rising mortality
Luo Y. / 2021 [114]	United States	From June 2020 to February 2021	No	Few cases or deaths / Second and third wave; high mortality
Majeed M.M. et al. / 2021 [115]	Pakistan	May 2020	No	First wave; high mortality
Martina S. et al. / 2021 [116]	Italy	May 2020	No	First wave; high mortality
Moffat, R.C. et al. / 2020 [117]	United States	May 2020	No	First wave; decreasing mortality
Moghadam M.G. et al. / 2021 [118]	Iran	From 21st to 27th June 2020	No	Few cases or deaths
Nardi G.M. et al. / 2021 [119]	Italy	From November 1st to December 30th 2020	No	Second wave; high mortality
Nazir M. et al. / 2021 [120]	Saudi Arabia	From June to July 2020	No	Second wave; rising mortality

Table S3. Continued.

First author / Publication year	Study location	Period of data collection	Vaccination process started (Yes/No)	Pandemic moment
Olivieri J.G. et al. / 2021 [121]	Spain	Strict confinement: from March 14th to May 21st 2020<br>Partial confinement: from May 25th to June 18th 2020	No	Strict confinement: first wave; high mortality<br>Partial confinement: few cases or deaths
Olszewska, A.; Rzymski, P. / 2020 [122]	Poland	2018 / From March 24th to April 30th 2020	No	Few cases or deaths
Papautsky E.L. et al. / 2021 [123]	United States	From April 5th to May 5th 2020	No	First wave; peak mortality
Pasiga, B.D. / 2020 [124]	Indonesia	From May 21st to June 13th 2020	No	Few cases or deaths
Peloso, R.M. et al. / 2020 [125]	Brazil	April 2020	No	First wave; low mortality
Phadraig C.M.G. et al. / 2021 [126]	Worldwide<br>Most from Europe (40.4%)	From 10th to 31st July 2020	No	Few cases or deaths
Pylińska-Dąbrowska, D. et al. / 2020 [127]	Poland	From November 2019 to September 2020	No	Pre-pandemic / First 6 months; low mortality
Quan S. et al. / 2021 [128]	China	From February 20th to March 5th 2020	No	First wave; high mortality
Samuel S.R. et al. / 2021 [129]	India	From March to June 2020	No	First wave; low mortality
Sari A.; Bilmez Z.Y. / 2021 [130]	Turkey	From August 1st to October 1st 2020	No	Second wave; low mortality
Tatar N.; Karabas A. / 2021 [131]	Turkey	From July to October 2020	No	Few cases or deaths / Second wave; low mortality
Vanka S. et al. / 2021 [132]	Saudi Arabia	2020	No	Unknown

Table S3. Continued.

First author / Publication year	Study location	Period of data collection	Vaccination process started (Yes/No)	Pandemic moment
Vohra P. et al. / 2021 [133]	India	From November 2020 to February 2021	Yes	First wave; low mortality
Wen Y.F. et al. / 2021 [134]	China	From February to March 2020	No	First wave; high mortality
Wu, Y. et al. / 2021 [135]	China	From 19th to 29th February 2020	No	First wave; high mortality
Xiong, X. et al. / 2020 [136]	China	From 20th to 22nd February 2020	No	First wave; high mortality
Yavan M.A. / 2021 [137]	Turkey	June 2020	No	Few cases or deaths

Table S4. Quality assessment of studies on dental professionals using the modified Newcastle-Ottawa Scale.

First author / Publication year	Selection			Comparability	Outcome			Score (*)
	Representativeness of the sample	Sample size	Non-respondents	Confounding factors	Assessment of the outcome I	Assessment of the outcome II	Statistical test	
Ahmed M.A. et al. / 2020 [15]	*			**	*	*	*	6
Ajwa N. et al. / 2020 [16]	*	*	*	*	*	**	*	8
Aldhuwayhi S. et al. / 2021 [17]	*			*	*	**	*	6
Alencar C. de M. et al. / 2021 [18]	*	*	*	*	*	**	*	8
Alkhalifah F.N. et al. / 2021 [19]	*			*	*	*	*	5
Aly M.M.; Elchaghaby M.A. / 2020 [20]	*	*	*	*	*	*		6
Amato A. et al. / 2021 [21]	*			**	*	*	*	6
Aurlene N. et al. / 2021 [22]	*	*	*	*	*	*		6
Balkaran R. et al. / 2021 [23]	*			*	*	*	*	5
Bellini P. et al. / 2020 [24]	*			*	*	**		5
Campos J.A.D.B. et al. / 2021 [25]	*			*	*	**	*	6
Çelik O.E.; Cansever İ.H. / 2021 [26]	*			**	*	*	*	6
Chakraborty T. et al. / 2020 [27]	*			**	*	**	*	7
Chaudhary F.A. et al. / 2021 [28]	*			**	*	**	*	7
Chen Y.; Li W. / 2021 [29]	*	*	*	**	*	**	*	9
Cheng H-C. et al. / 2021 [30]	*			**	*	*	*	6
Collin V. et al. / 2021 [31]	*			*	*	**	*	6
Consolo U. et al. / 2020 [32]	*	*	*	*	*	**	*	8
Cotrin P. et al. / 2020 [33]	*	*	*	*	*	*	*	7
De Stefani A. et al. / 2020 [34]	*			*	*	*	*	5
Dreher A. et al. / 2021 [35]	*			**	*	**	*	7
Estrich C.G. et al. / 2021 [36]	*		*	*	*	**	*	7
Fairozekhan A.T. et al. / 2021 [37]	*		*	*	*	*	*	6
Ortega-López M.F. et al. / 2021 [38]		*		**	*	**	*	7
Gasparro R. et al. / 2020 [39]	*			**	*	**	*	7
Humphris G. et al. / 2021 [40]	*	*	*	*	*	**	*	8
Iorga M. et al. / 2021 [41]	*			*	*	**	*	6
Kale P. et al. / 2021 [42]	*		*	*	*	**	*	7
Kamal A.T. et al. / 2021 [43]	*	*	*	**	*	**	*	9

(*) = <5 : Unsatisfactory quality / 5 · 6 : Satisfactory quality / 7 · 8 : Good quality / 9 · 10 : Very Good quality

Table S4. Continued.

First author / Publication year	Selection			Comparability	Outcome			Score (∗)
	Representativeness of the sample	Sample size	Non-respondents	Confounding factors	Assessment of the outcome I	Assessment of the outcome II	Statistical test	
Kamran R. et al. / 2021 [44]	∗			∗	∗	∗	∗	5
Karayürek F. et al. / 2021 [45]	∗			∗	∗	∗	∗	5
Karobari M.I. et al. / 2021 [46]	∗		∗	∗	∗	∗	∗	6
Khader Y. et al. / 2020 [47]	∗			∗	∗	∗	∗	5
Kirli M.C.; Kirli U. / 2021 [48]			∗	∗	∗	∗∗	∗	6
Labban N. et al. / 2021 [49]	∗	∗	∗	∗	∗	∗∗	∗	8
Mahdee A.F. et al. / 2020 [50]	∗	∗	∗	∗	∗	∗	∗	7
Martina S. et al. / 2020 [51]	∗			∗∗	∗	∗∗	∗	7
Mekhemar M. et al. / 2021 [52]	∗	∗	∗	∗∗	∗	∗∗	∗	9
Mekhemar M. et al. / 2021 [53]	∗	∗	∗	∗∗	∗	∗∗	∗	9
Mijiritsky E. et al. / 2020 [54]	∗			∗∗	∗	∗∗	∗	7
Mishra S. et al. / 2020 [55]	∗	∗	∗	∗	∗	∗∗	∗	8
Moraes R.R. et al. / 2021 [56]	∗	∗	∗	∗∗	∗	∗	∗	8
Mulla S. et al. / 2020 [57]	∗			∗	∗	∗∗	∗	6
Nagarajappa R. et al. / 2021 [58]	∗			∗∗	∗	∗∗	∗	7
Nair A.K.R. et al. / 2020 [59]	∗			∗∗	∗	∗∗	∗	7
Nallamothu R. et al. / 2021 [60]	∗	∗	∗	∗	∗	∗	∗	7
Olivieri J.G. et al. / 2021 [61]			∗	∗	∗∗	∗∗	∗	7
Owen C. et al. / 2021 [62]	∗			∗	∗	∗		4
Özarslan M.; Caliskan S. / 2021 [63]	∗	∗	∗	∗	∗	∗∗	∗	8
Pai S. et al. / 2021 [64]	∗	∗	∗	∗∗	∗	∗	∗	8
Popatrao Patil A. et al. / 2021 [65]	∗			∗	∗	∗		4
Peixoto K.O. et al. / 2021 [66]	∗			∗∗	∗	∗∗	∗	7
Plessas A. et al. / 2021 [67]	∗			∗	∗	∗		4
Prajapati A.S. et al. / 2021 [68]	∗			∗	∗	∗	∗	5
Prasetyo Y.T. et al. / 2021 [69]	∗			∗∗	∗	∗	∗	6
Ramesh M. et al. / 2020 [70]	∗	∗	∗	∗	∗	∗	∗	7
Ranka M.S.; Ranka S.R. / 2021 [71]		∗	∗	∗	∗	∗∗	∗	7
Salehiniya H.; Abbaszadeh H. / 2021 [72]	∗			∗∗	∗	∗∗	∗	7
Sandhu B.K. et al. / 2021 [73]	∗			∗	∗	∗		4
Sarapultseva M. et al. / 2021 [74]				∗∗	∗	∗∗	∗	6

(∗) = <5 : Unsatisfactory quality / 5 · 6 : Satisfactory quality / 7 · 8 : Good quality / 9 · 10 : Very Good quality

Table S4. Continued.

First author / Publication year	Selection			Comparability	Outcome			Score (∗)
	Representativeness of the sample	Sample size	Non-respondents	Confounding factors	Assessment of the outcome I	Assessment of the outcome II	Statistical test	
Sarialioglu Gungor A. et al. / 2021 [75]	∗	∗	∗	∗	∗	∗	∗	7
Schlenz M.A. et al. / 2021 [76]	∗			∗∗	∗	∗	∗	6
Schmidt J. et al. / 2021 [77]		∗	∗	∗	∗	∗		5
Serota K.S. et al. / 2021 [78]	∗			∗∗	∗	∗∗	∗	7
Shacham M. et al. / 2021 [79]	∗			∗∗	∗	∗∗	∗	7
Shetty A. et al. / 2020 [80]	∗	∗	∗	∗	∗	∗	∗	7
Singh Y.P. / 2021 [81]	∗			∗	∗	∗	∗	5
Suryakumari V.B.P. et al. / 2020 [82]	∗			∗∗	∗	∗	∗	6
Tao J. et al. / 2021 [83]	∗	∗	∗	∗∗	∗	∗∗	∗	9
Tokuc B.; Coskunses F.M. / 2020 [84]	∗			∗	∗	∗	∗	5
Turska-Szybka A. et al. / 2021 [85]	∗			∗	∗	∗	∗	5
Tysiąc-Miśta M.; Dziedzic A. / 2020 [86]	∗			∗	∗	∗	∗	5
Uhlen M.M. et al. / 2021 [87]	∗			∗	∗	∗	∗	5
Upadhyay N. et al. / 2021 [88]	∗	∗	∗	∗∗	∗	∗	∗	8
Uziel N. et al. / 2021 [89]	∗			∗	∗	∗∗	∗	6
Wajeeh S. et al. / 2021 [90]	∗	∗	∗	∗∗	∗	∗	∗	8
Yilmaz H.N.; Ozbilen E.O. / 2020 [91]	∗			∗	∗	∗∗	∗	6
Yılmaz M. et al. / 2021 [92]	∗		∗	∗∗	∗	∗∗	∗	8
Zeidi I.M.; Zeidi B.M. / 2021 [93]		∗	∗	∗∗	∗	∗∗	∗	8
Zhao S. et al. / 2020 [94]	∗			∗∗	∗	∗∗	∗	7

(∗) = <5 : Unsatisfactory quality / 5 · 6 : Satisfactory quality / 7 · 8 : Good quality / 9 · 10 : Very Good quality

Table S5. Quality assessment of studies on dental patients using the modified Newcastle-Ottawa Scale.

First author / Publication year	Selection			Comparability	Outcome			Score (*)
	Representativeness of the sample	Sample size	Non-respondents	Confounding factors	Assessment of the outcome I	Assessment of the outcome II	Statistical test	
Abdulkareem A.A. et al. / 2021 [95]	*	*		**	*	*	*	7
Arqub S.A. et al. / 2021 [96]	*		*	*	*	**	*	7
Arslan I.; Aydinoğlu S. / 2021 [97]	*	*	*	*	*	**	*	8
Ashraf A. et al. / 2021 [98]	*			**	*	*	*	6
Azevedo Machado B. et al. / 2021 [99]	*	*		**	*	**	*	8
Berberoğlu B. et al. / 2021 [100]				*	*	**	*	5
Blumer S. et al. / 2021 [101]	*			**	*	*	*	6
Bustati N.; Rajeh N. / 2020 [102]	*	*	*	*	*	*		6
Campagnaro R. et al. / 2020 [103]	*	*	*	*	*	*	*	7
Carrillo-Diaz M. et al. / 2021 [104]			*	**	*	**	*	7
Cotrin P. et al. / 2020 [105]			*	*	*	**	*	6
Di Giacomo P. et al. / 2021 [106]				*	*	**	*	5
Farsi D.; Farsi N. / 2021 [107]	*			**	*	*	*	6
Folayan M.O. et al. / 2021 [108]	*	*	*	**	*	**	*	9
Gallegati S. et al. / 2021 [109]	*			**	*	*	*	6
González-Olmo M.J. et al. / 2021 [110]	*		*	**	*	**	*	8
González-Olmo M.J. et al. / 2020 [111]				*	*	**	*	5
Hajek A. et al. / 2021 [112]	*		*	**	*	*	*	7
Ibrahim M.S. et al. / 2021 [113]	*	*	*	**	*	**	*	9
Luo Y. / 2021 [114]	*			**	*	**	*	7
Majeed M.M. et al. / 2021 [115]	*	*	*	*	*	*	*	7
Martina S. et al. / 2021 [116]	*			**	*	**	*	7
Moffat R.C. et al. / 2020 [117]	*			**	*	*	*	6
Moghadam M.G. et al. / 2021 [118]		*	*	*	*	**	*	7
Nardi G.M. et al. / 2021 [119]	*			**	*	*	*	6
Nazir M. et al. / 2021 [120]	*	*	*	**	*	**	*	9
Olivieri J.G. et al. / 2021 [121]	*	*	*	*	*	**	*	8
Olszewska A.; Rzymski P. / 2020 [122]		*		*	*	*	*	5
Papautsky E.L. et al. / 2021 [123]	*			**	*	*	*	6
Pasiga B.D. / 2020 [124]	*			*	*	*	*	5
Peloso R.M. et al. / 2020 [125]		*	*	*	*	*	*	6

(*) = <5 : Unsatisfactory quality / 5 · 6 : Satisfactory quality / 7 · 8 : Good quality / 9 · 10 : Very Good quality

Table S5. Continued.

First author / Publication year	Selection			Comparability	Outcome			Score (∗)
	Representativeness of the sample	Sample size	Non-respondents	Confounding factors	Assessment of the outcome I	Assessment of the outcome II	Statistical test	
Phadraig C.M.G. et al. / 2021 [126]	∗			∗	∗	∗	∗	5
Pylińska-Dąbrowska D. et al. / 2020 [127]				∗	∗	∗∗	∗	5
Quan S. et al. / 2021 [128]	∗			∗∗	∗	∗∗	∗	7
Samuel S.R. et al. / 2021 [129]	∗	∗	∗	∗∗	∗	∗∗	∗	9
Sari A.; Bilmez Z.Y. / 2021 [130]	∗	∗	∗	∗∗	∗	∗∗	∗	9
Tatar N.; Karabas A. / 2021 [131]				∗	∗	∗	∗	4
Vanka S. et al. / 2021 [132]			∗	∗	∗	∗	∗	5
Vohra P. et al. / 2021 [133]	∗			∗	∗	∗	∗	5
Wen Y.F. et al. / 2021 [134]				∗∗	∗	∗∗	∗	6
Wu Y. et al. / 2021 [135]	∗			∗∗	∗	∗∗	∗	7
Xiong X. et al. / 2020 [136]				∗∗	∗	∗∗	∗	6
Yavan M.A. / 2021 [137]			∗	∗	∗	∗∗	∗	6

(∗) = <5 : Unsatisfactory quality / 5 · 6 : Satisfactory quality / 7 · 8 : Good quality / 9 · 10 : Very Good quality
